# DPP4 inhibition affects metabolism and inflammation associated pathways in hiPSC-derived steatotic HLCs

**DOI:** 10.3389/fcell.2026.1686556

**Published:** 2026-02-26

**Authors:** Christiane Loerch, Wasco Wruck, Annika Wittich, Rabea Hokamp, Julian Reiss, Ole Pless, James Adjaye, Nina Graffmann

**Affiliations:** 1 Institute for Stem Cell Research and Regenerative Medicine, Medical Faculty and University Hospital Düsseldorf, Heinrich Heine University, Düsseldorf, Germany; 2 Fraunhofer Institute for Translational Medicine and Pharmacology ITMP, Discovery Research ScreeningPort, Hamburg, Germany; 3 University College London, EGA Institute for Women`s Health, Zayed Center for Research into Rare Diseases in Children (ZGR), London, United Kingdom

**Keywords:** diabetes, dipeptidyl peptidase 4, hepatocyte-like cells, human induced pluripotent stem cells, MAFLD/MASLD, vildagliptin

## Abstract

**Introduction:**

Metabolic dysfunction-associated steatotic liver disease (MAFLD) has a high prevalence and high comorbidity for other diseases. Due to the complexity of this multifactorial disease, therapy options are still rather limited. We employed an *in vitro* pluripotent stem cell-based model to decipher potential disease-associated molecular pathways and study the mode of action of prospective drugs. Dipeptidyl peptidase 4 (DPP4) or cluster of differentiation 26 (CD26) is involved in inflammation, infections, immune disorders, type 2 diabetes, kidney disease, and cancer.

**Methods:**

We induced the steatosis phenotype in human induced pluripotent stem cell (iPSC)-derived hepatocyte-like cells (HLCs) by oleic acid (OA) feeding and confirmed regulation of clinically relevant pathways by NGS-based global transcriptomic analyses. Analysis of the secretome of steatotic HLCs revealed DPP4 as a potential key mediator of the disease. To further elucidate its role in the development of MAFLD, we inhibited DPP4 activity with vildagliptin (VILDA) and analyzed the global transcriptomic changes and specific gene and protein gene expression of steatosis-associated genes with and without DPP4 inhibition.

**Results:**

MAFLD-associated pathways such as PPAR and TNF signaling were differentially regulated in hiPSC-derived steatotic HLCs. We found increased hepatic DPP4 activity and secretion upon OA feeding. Gene expression of fatty acid and purine metabolism and inflammation-associated pathways was regulated upon DPP4 inhibition.

**Discussion:**

Our HLC model confirmed the association of DPP4 with metabolism and inflammation, which foster the development of MAFLD. Inhibiting DPP4 activity with VILDA partially relieved the steatotic phenotype on a global transcriptomic level.

**Impact and implications:**

Given the difficulties of identifying suitable anti-MAFLD drugs, novel model systems are urgently needed. Our *in vitro* HLC-model reproduced the DPP4-dependent aspects of the disease and responded positively to VILDA treatment. Further elucidation of the role of DPP4 in the etiology of MAFLD and other diseases is warranted.

## Graphical Abstract

**Figure:**
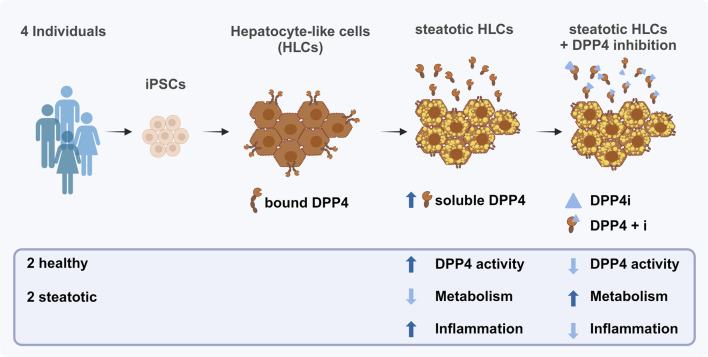

## 1 Introduction

Steatotic liver diseases are an increasing health burden for industrialized countries all over the globe (Angulo, 2007; Eslam et al., 2020; Henry et al., 2022; Teng et al., 2023). The early, reversible stages involve steatosis and steatohepatitis, resulting in fibrosis, while nascent cirrhosis and hepatocellular carcinoma are non-reversible and have life-threatening implications. The primary causes for steatosis are elevated fatty acid flux from adipose tissue, a high-fat diet, or elevated blood glucose levels. Thus, obesity and type 2 diabetes mellitus (T2DM) are direct diagnostic criteria for metabolic dysfunction-associated fatty/steatotic liver disease (MAFLD/MASLD) (Eslam et al., 2020). MAFLD is a complex, multisystem disease, with serious implications on the whole body, including cardiovascular disease and chronic kidney disease (Barrera et al., 2024; Zhao et al., 2024). Moreover, its multifactorial characteristic and tissue heterogeneity are not only found in the clinic but are also observed in the molecular response to steatosis (Wruck et al., 2015; Graffmann et al., 2016; Graffmann et al., 2021). These factors complicate research, and, to date, resmetirom is the only FDA-approved drug for treating MASLD (Zhao et al., 2024; Beygi et al., 2024; Lara-Romero and Romero-Gómez, 2024).

The dipeptidyl peptidase 4 (DPP4) family consists of several serine proteases, the most prominent one being DPP4, with catalytic activity for various substrates (Waumans et al., 2015). The major function of DPP4 is incretin regulation via degradation of glucagon-like peptide 1 (GLP-1) and glucose-dependent insulinotropic polypeptide (GIP1) (Deacon, 2019). However, it also cleaves chemotactic peptides with implications in inflammatory response (Mulvihill and Drucker, 2014; Trzaskalski et al., 2020). DPP4 is considered a hepatokine, which is upregulated in metabolic liver disease, and is a driver of inflammation (Trzaskalski et al., 2020). Furthermore, DPP4 is involved in various etiologies, such as immune disorders, fibrosis, and cancer (de Meester et al., 2003). Gliptins—potent DPP4 inhibitors—are used for the treatment of T2DM, prolonging the postprandial incretin response (Trzaskalski et al., 2020; Ahrén et al., 2004; Ghorpade et al., 2018; Ohm et al., 2023). Vildagliptin (VILDA) is a major inhibitor of DPP4 activity; however, it can also inhibit the activities of other DPPs, especially DPP8 and 9 and FAP (Waumans et al., 2015; Burkey et al., 2008). In addition to glycemic control, it showed protective effects on hepatocytes by reducing the hepatic triglyceride load and aminotransferase levels (Hussain et al., 2016). Furthermore, inflammatory pathways were regulated, potentially regulating the traits toward steatohepatitis, thus benefiting disease progression (Khalil et al., 2020; Hendawy et al., 2022; Macauley et al., 2015). Nevertheless, since these experiments were conducted mainly in rodents or hepatoma cell-based models, a relevant human hepatic steatosis model is necessary.

In this study, we differentiated human patient-derived induced pluripotent stem cells (iPSCs) into hepatocyte-like cells (HLCs) by using our previously published, efficient 2D differentiation protocol (Loerch et al., 2024). We induced the steatosis phenotype and provide insights into the hepatocyte-specific contribution to the disease and the potential role of DPP4 in the interplay between metabolism and inflammation.

## 2 Materials and methods

### 2.1 Human-induced pluripotent stem cell (iPSC) culture and hepatocyte-like cell (HLC) differentiation

The use of iPSC lines for this study was approved by the ethics committee of the medical faculty of Heinrich-Heine-University (5013 and 5704). iPSCs were cultivated on Matrigel (Corning) coated 6-well plates with the StemMACs medium (Miltenyi) changed daily. Once iPSCs attained 90% confluency, they were passaged by the addition of PBS w/o magnesium or calcium (PBS^−/−^) (Gibco) and incubated for approximately 3 min at room temperature (RT). Colonies were detached from the surface with a cell scraper and centrifuged at 40 x g for 3 min. The pellet was carefully resuspended, and clumps of colonies were seeded at a ratio of 1:6. The used iPSC lines are shown in Table 1.

**TABLE 1:** iPSC lines used in this study.

iPSC line	Sex	Age (years)	Disease	Source	Ref.
Cntrl 1	Male	50	Healthy	Urine-derived renal progenitor cells	Bohndorf et al. (2017)
Cntrl 2	Female	19	Healthy	Dermal fibroblasts	Kawala et al. (2016a)
Stea 1	Male	61	High-grade steatosis	Dermal fibroblasts	Kawala et al. (2016b)
Stea 2	Female	58	High-grade steatosis	Dermal fibroblasts	Graffmann et al. (2018)

iPSCs were differentiated according to our previously published protocol (Loerch et al., 2024). In brief, 1.04 x10^5^ iPSCs/cm^2^ were seeded onto Matrigel-coated dishes. Definitive endoderm (DE) was induced by 1–3 days of 2.5 µM CHIR99021 (Stemgent) and 3–5 days of 100 ng/mL Activin A (Peprotech) in RPMI medium. Hepatic endoderm (HE) medium was fed for 4 days, and 1% DMSO was added with medium changes every day. HLC medium was fed for 12–15 days, with medium changes every other day. 1 μM insulin (Sigma-Aldrich), 10 ng/mL hepatocyte growth factor (HGF) (Peprotech), 25 ng/mL dexamethasone (Dex) (Sigma-Aldrich), and 20 ng/mL recombinant human oncostatin M (rhOSM209a.a) (ImmunoTools) were freshly added to the medium.

### 2.2 Immunocytochemistry

Cells were washed with PBS^−/−^, fixed with 4% PFA for 10 min at RT, and washed 3x with PBS^−/−^. For intracellular staining, the cells were permeabilized with 0.5% Triton-X-100 (Sigma-Aldrich) in PBS^−/−^ for 10 min at RT and blocked with 3% BSA in PBS^−/−^ for 1 h at RT. After incubation with primary antibodies at respective dilutions (supplementary material, Supplementary Table S2) overnight at 4 °C, unbound antibodies were washed off 3x with PBS^−/−^. Secondary antibodies against the respective host IgG were incubated for 1 h at RT and washed 3x with PBS^−/−^. Confocal or epifluorescence microscopy was performed using a LSM 700 microscope (Zeiss), and images were processed with ZEN software (Zeiss).

### 2.3 Quantification of immunoassayed markers

To quantify the developmental stages during differentiation, 5 .tiff images of each cell line and condition were analyzed for the respective markers. OCT4 as pluripotency marker, SOX17 for definitive endoderm, HNF4a for hepatic endoderm and ALB, HNF4 for hepatocyte-like cells. An image analysis pipeline was established using different building blocks. Nuclei were identified based on the Hoechst channel using the “Find Nuclei” block. The intensity and morphology parameters were calculated with the “Calculate Intensity Properties” and “Calculate Morphology Properties” blocks, respectively. Based on these properties, a final nucleus population was selected using the “Select Population” function, removing dead cells and incorrectly identified nuclei. Within this final population of nuclei, channel intensities for specific nuclear markers (SOX17, HNF4a, and OCT4) were calculated using the “Calculate Intensity Properties” block. Using individual intensity thresholds for each cell line, positive cell populations for each marker were defined with the “Select Population” function. For ALB staining, the “Find Cytoplasm” block identified the cell area surrounding the nuclei. Inside these areas, ALB intensities were measured with the “Calculate Intensity Properties” function. Again, thresholds tailored to each cell line were used to define ALB-positive cells with the “Select Population” function. The percentage of positive cells for each marker was calculated as (number of positive cells/final nuclei) * 100 and expressed as means ± standard deviation (SD).

### 2.4 Quantitative reverse transcription PCR (qRT-PCR)

RNA was isolated using the Direct-zol RNA isolation kit (Zymo Research), following the manufacturer’s instructions. 500 ng of RNA was reverse-transcribed to cDNA using the TaqMan reverse transcription kit (Life Technologies). qRT-PCR was performed using the VIIA7 machine and the power SYBR green master mix (all Life Technologies). For each donor, RNA from three preparations was analyzed in triplicates. For the characterization of HLCs, the CT values were normalized to the housekeeping gene *RPLP0* and then normalized to the expression detected in iPSCs, which was set to 0. Expression of mRNA was presented as log2-fold-change and shown as the mean ±standard error of the mean (SEM) (primer sequences are provided in Supplementary Table S1).

### 2.5 Western blot

Cells were lysed in RIPA buffer containing protease and phosphatase inhibitors (all Sigma-Aldrich). An amount of 15–30 µg of proteins was separated on NuPAGE 4%–12% Bis–Tris protein gels (Life Technologies) and wet-blotted onto 0.45 µm nitrocellulose membranes (Amersham). After blocking with 5% non-fat milk (ROTH) in TBS-T buffer, the membranes were incubated with the respective primary antibodies (Supplementary Table S2) overnight at 4 °C. After washing 3x with TBS-T buffer, fluorescence-labeled secondary antibodies (LICOR) against the host IgG were incubated for 1 h at RT at a 1:10,000 dilution. Unbound antibodies were washed off with TBS-T buffer, and the fluorescence signal was detected at 680 nm and 800 nm using the ChemiDoc MP Imaging system (Bio-Rad). Quantification for three preparations of each treatment for Cntrl 1 and 2 was performed using the Image Lab 6.0.1 software with lane background subtraction using disk size 1 and is presented as means ± SD.

### 2.6 Cytochrome P450 activity measurement

P450-Glo™ CYP3A4 and CYP2D6 assays (Promega) were used to measure cytochrome P450 activity for each donor. Cells were incubated with 3 µM luciferin-IPA or 10 µM luciferin-ME EGE, respectively, in William’s E Medium (Gibco) for 1 h at 37 °C. After incubation with the detection reagent, luminescence was measured in technical triplicates with a luminometer (Lumat LB 9507, Berthold Technologies). For each donor, HLCs from one experiment were used for analysis in triplicates. The results are shown as means ± SD.

### 2.7 Steatosis induction by OA feeding and vildagliptin (VILDA) treatment

Oleic acid (OA) (Calbiochem) was bound to 14% (w/v) fatty acid-free BSA (ROTH) in 0.1 M TRIS at pH 8.0 for 1 h at 37 °C and stored at 4 °C. After testing different concentrations and time periods of OA treatment, we selected 400 µM OA for 7 days to induce steatosis. From days 15–17 onward of HLC differentiation, the cells were fed with complete HLC medium, supplemented with 400 µM OA or the respective volume of TRIS-BSA as mock treatment. The medium was changed every other day for 7 days. A final concentration of 30 µM VILDA (Sigma-Aldrich) dissolved in DMSO was fed to the cells after 48 h OA-/mock-induction for 5 days with medium changes every other day.

### 2.8 Next-generation sequencing and analysis of deep sequencing data

For each donor and condition, RNA was isolated from three preparations. 3′RNA-Seq was performed on a NextSeq2000 sequencing system (Ilumina) at the core facility Biomedizinisches Forschungszentrum Genomics and Transcriptomics laboratory (BMFZ-GTL) of Heinrich-Heine-University Duesseldorf. HISAT2 (version 2.1.0) software (Kim et al., 2015) was employed to align the fastq files received from the BMFZ-GTL core facility against the GRCh38 genome. For detailed description of the integration of the data, please refer to the Supplementary Material methods section.

### 2.9 GO and pathway analysis

Subsets of genes expressed exclusively in one condition in the Venn diagram analysis and up- and down-regulated genes according to the criteria for differentially expressed genes (limma test, p-value <0.05, and fold change >1.5) were subjected to over-representation analysis of Gene Ontology (GO) and KEGG (Kyoto Encyclopedia of Genes and Genomes) pathways (Kanehisa et al., 2017). The hypergeometric test built-in in the R base package was used for over-representation analysis of KEGG pathways, which had been downloaded from the KEGG database in February 2023. The GOstats R package (Falcon and Gentleman, 2007) was utilized to determine the over-represented GO terms. The most significant GO terms and KEGG pathways are displayed in dotplots via the R package ggplot2 (Wickham, 2009).

### 2.10 Enzyme-linked immunosorbent assay (ELISA)

Secreted DPP4 was detected from the supernatants 48 h after feeding using the human DPP4/CD26 DuoSet ELISA (R&D Systems), as described by the manufacturer. The optical density was measured using the EPOCH2 spectrophotometer (BioTek) at 450 nm with wavelength correction at 540 nm. 4-PL curve fitting was performed to calculate the concentrations. For each donor and condition, supernatant was collected from three preparations, analyzed in triplicates, and depicted as means ± SD.

### 2.11 Enzymatic activity assay

DPP4 activity was measured in OA-/VILDA-treated HLCs using the Dipeptidyl peptidase IV Activity Assay Kit (Fluorometric) (Abcam), following the manufacturer’s instructions. The fluorescence signal was measured on a spectrophotometer (Tecan) at Ex/Em = 360/460 nm. For each donor and condition, the supernatant was collected from three preparations, analyzed in duplicates, and depicted as means ±SD.

### 2.12 Cytokine array

Supernatants of three biological replicates were harvested 24 h after changing the medium. Proteome Profiler Human XL Cytokine array (R&D Systems) analysis was performed following the manufacturer’s protocol, and signals were detected using the Fusion FX instrument (PeqLab). Analysis and quantification were performed using the FIJI/ImageJ software (Schneider et al., 2012) and the Microarray Profile plugin by Bob Dougherty and Wayne Rasband (https://www.optinav.info/MicroArray_Profile.htm, accessed on 21 December 2022). For details of the image analysis and follow-up normalization in the R/Bioconductor environment (Schneider et al., 2012), we refer to the description in our previous publication (Wruck et al., 2022). Cytokines were considered differentially expressed if they satisfied the following criteria: detection p-value <0.05 in at least one condition, fold change >1.2, and limma-p-value <0.05. The function heatmap.2 from the gplots package (Warnes et al., 2015) and the R-builtin function barplot were applied for heatmap and bar plots.

## 3 Results

### 3.1 iPSC-derived hepatocyte-like cell (HLC) differentiation from four individuals

**FIGURE 1:**
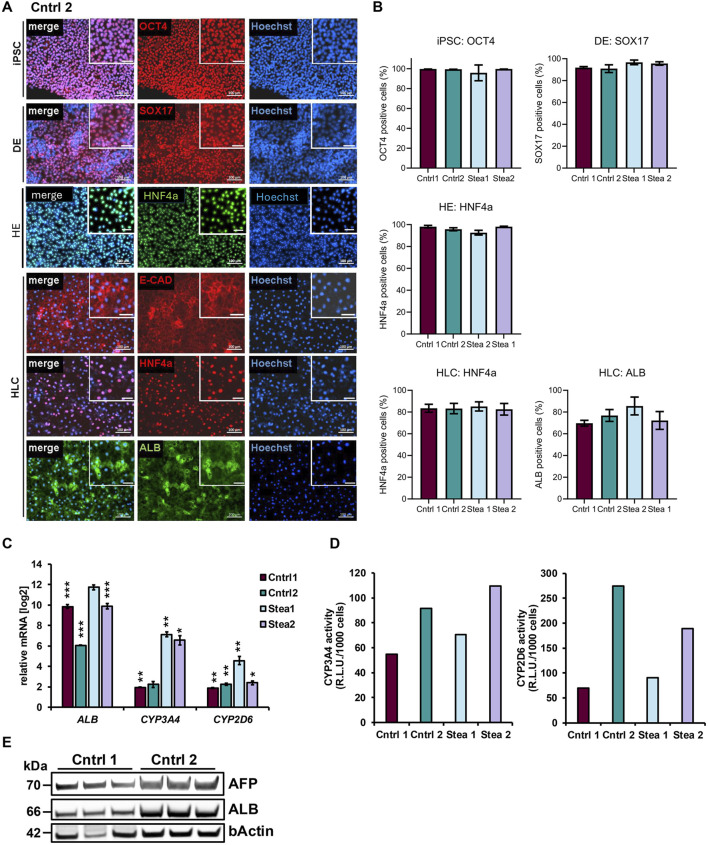
Characterization of HLCs. **(A)** Representative immunocytochemistry of cell line Cntrl 2 during differentiation showing the respective markers OCT4 in iPSCs, SOX17 in definitive endoderm (DE), and HNF4a in hepatic endoderm (HE). The epithelial markers E-CAD, HNF4a, and ALB are shown for hepatocyte-like cells (HLCs). Scale bars = 100 µm. **(B)** Protein expression in respective differentiation states of the four cell lines. **(C)** gene expression levels of *ALB* and cytochrome P450 family members *CYP3A4* and *CYP2D6* in HLCs from four donors relative to the iPSC-stage that is equivalent to 0, shown as means ±SEM, respectively, normalized to the housekeeping gene *RPLP0*. Two-tailed unpaired Students’ T-test was performed to calculate significances (*p < 0.05, **p < 0.01, and ***p < 0.001). **(D)** Representative cytochrome P450 3A4 (CYP3A4) and 2D6 (CYP2D6) activities of HLCs from four donors are depicted as relative light units (R.L.U.) per 1,000 cells. **(E)** WB of AFP, ALB, and b-actin in HLCs derived from two donors. Uncropped full-length blots can be found in Supplementary Figure S12.

iPSCs derived from four individuals, including two healthy controls (Cntrl 1 and Cntrl 2) (Bohndorf et al., 2017; Kawala et al., 2016a) and two steatosis patients (Stea 1 and Stea 2) (Kawala et al., 2016b; Graffmann et al., 2018) (Table 1), were differentiated into HLCs following our recently published protocol (Loerch et al., 2024). Representative immunocytochemistry of Cntrl 2 shows the expression of octamer binding transcription factor 4 (OCT4) in iPSCs, SRY-box transcription factor 17 (SOX17) in definitive endoderm (DE), and hepatocyte nuclear factor 4 alpha (HNF4a) in hepatic endoderm (HE) (Figure 1A). HLCs were stained for the epithelial marker E-cadherin (E-CAD), HNF4a, and albumin (ALB) (Figure 1A). Representative microphotographs of the other cell lines are provided in Supplementary Figures S1, S2A–C. Protein expression in the respective differentiation stages was quantified for each cell line. All four iPSC lines were >95% positive for OCT4. During differentiation, more than 90% of the cells adopted DE and HE fate, as shown by SOX17 and HNF4a expression, respectively. In the HLC stage, at least 80% of the cells were positive for HNF4a, while the expression of ALB was more variable, ranging from 69% in Cntrl 1 to 86% in Stea 2 (Figure 1B). HLCs showed significant increase in gene expression levels of the HLC-markers *ALB* and the cytochrome P450 family members *CYP3A4* and *CYP2D6* in comparison to the iPSC stage (Figure 1C). Representative gene expression of *OCT4* and *SOX17* in DE, along with *AFP* in the HE stage, are provided in Supplementary Figure S2D. HLCs’ functionality was confirmed by measuring CYP3A4 and CYP2D6 activity for the four cell lines (Figure 1D). Protein expression levels of AFP and ALB are shown in comparison to the housekeeping protein beta-actin (b-Actin) in HLCs derived from Cntrl 1 and Cntrl 2 (Figure 1E).

### 3.2 Oleic acid induces the steatosis phenotype in HLCs

**FIGURE 2:**
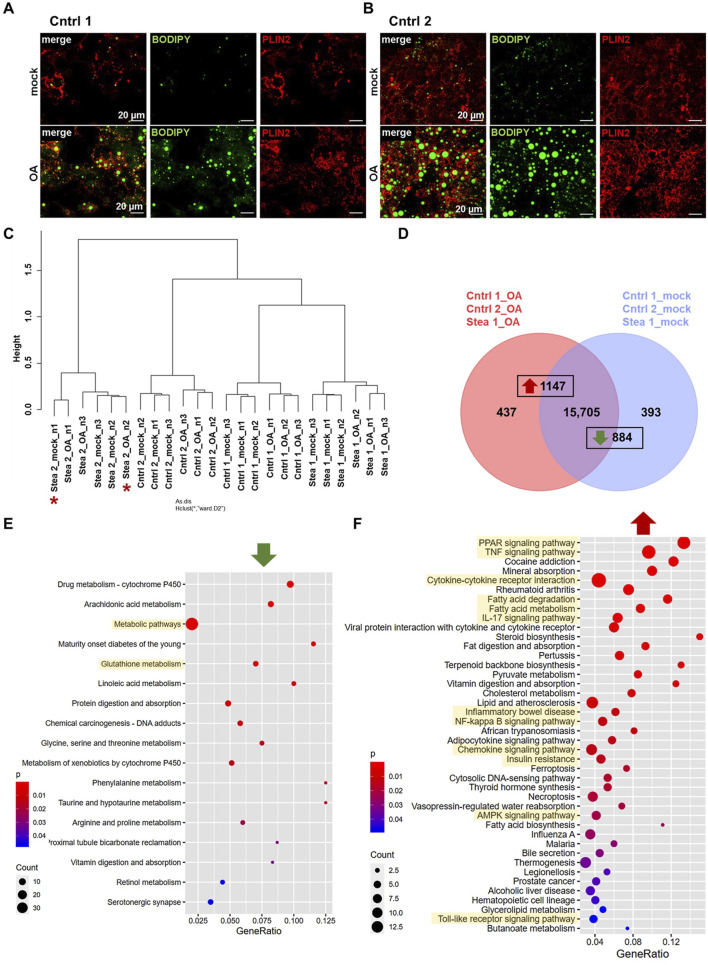
Confirmation of the steatosis phenotype in HLCs. **(A,B)** Representative immunofluorescence and BODIPY 493/503 staining of HLCs derived from two donors treated with 400 µM OA and respective control (mock) for 7 days; PLIN2 is shown in red, and fatty acids are stained in green. Scale bars represent 20 µm. **(C)** Hierarchical cluster dendrogram of global transcriptomic changes upon OA treatment of HLCs derived from four donors. Clustering of gene expression in Stea 2 deviating from the treatment is marked by red asterisks (correlation can be found in Supplementary Data Sheet S2). **(D)** Venn diagram of gene expression from OA-treated HLCs derived from three donors indicating 437 solely expressed genes upon OA treatment, 393 solely expressed genes upon mock treatment, and 15,705 genes expressed in common. Among exclusively and commonly expressed genes 1,147 and 834 genes were significantly up- and down-regulated, respectively. **(E,F)** Dot plots of KEGG-associated pathway analysis of significantly down **(E)**- and up **(F)**-regulated genes (gene lists are provided in Supplementary Data Sheet S2).

To induce the steatosis phenotype in iPSC-derived HLCs, we treated HLCs of all four cell lines on days 15–17 of differentiation with 400 µM OA for 7 days. After OA induction, we detected the formation of perilipin-2 (PLIN2)-coated lipid droplets by immunocytochemistry in all cell lines (Figures 2A,B; Supplementary Figure S3). Interestingly, from a visual impression, it appeared that Cntrl 1 showed less lipid droplets than Cntrl 2, indicating a cell line-specific difference in the build-up of lipid droplets. In accordance with previous findings, we did not detect a disease-specific difference in the lipid load with (w) or without (w/o) OA; instead, a cell-line-specific effect was observed (Graffmann et al., 2021). Previous findings indicated distinct gene expression profiles in response to OA treatment were related to the steatosis background of HLCs. To put these observations in perspective, we analyzed the global transcriptomic changes upon OA induction.

**FIGURE 3:**
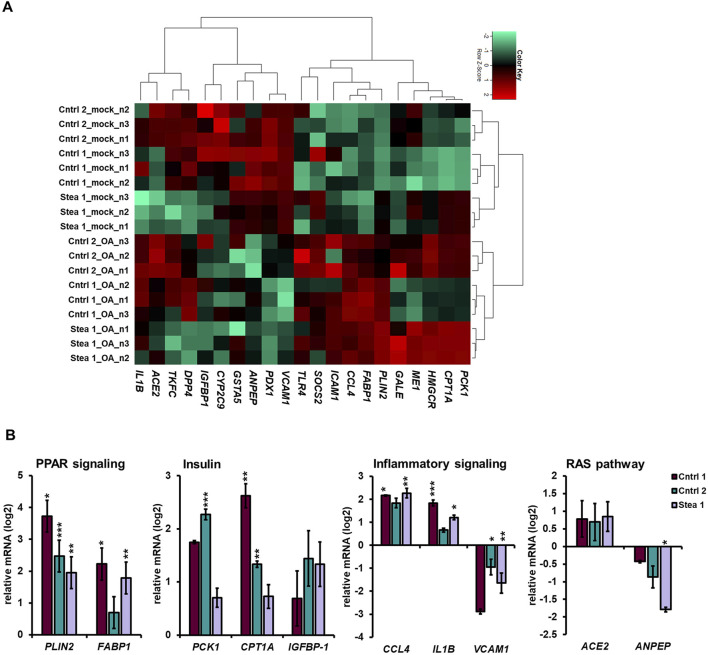
OA treatment induces differential gene expression. **(A)** Pearson’s correlation heatmap analysis of the gene expression of members of OA-dysregulated pathways such as fatty acid metabolism (*PLIN2* and *FABP1*), metabolic pathways/glutathione metabolism (*ANPEP* and *GSTA5*), and insulin resistance (*PCK1*, *CPT1A*, *IGFBP1*, *SERPINA7*, and *PDX1*). **(B)** qRT-PCR analysis of the mRNA expression of *PLIN2*, *FABP1, PCK1*, C*PT1A*, *IGFBP1*, *CCL4*, *IL1B, VCAM1*, *ACE2*, and *ANPEP* relative to mock-treated HLCs from three donors, shown as means± SEM, normalized to the housekeeping gene *RPLP0* and relative to the gene expression under the mock condition. Two-tailed unpaired Student’s T-test was performed to calculate significances (*p < 0.05, **p < 0.01, and ***p < 0.001).

RNA-seq was performed, and the results revealed gene expression clustering according to the treatment and based on the genetic background of each cell line (Figure 2C, Supplementary Figure S2). However, the gene expressions of two samples of HLCs derived from cell line Stea 2 did not cluster according to the treatment (Figure 2C, red asterisks). To prevent misinterpretation due to the genetic background of this cell line, we excluded Stea 2 in the subsequent analysis. We identified 15,705 genes expressed in common in both conditions in cell lines Cntrl 1, Cntrl 2, and Stea 1, while 437 genes were exclusively expressed under OA in comparison to 393 genes solely expressed under mock treatment. Combining the exclusively expressed genes and genes expressed in common, we found 1,147 significantly upregulated genes and 884 significantly downregulated genes (Figure 2D) after OA treatment. KEGG-associated pathway analysis revealed that genes of the glutathione pathway and metabolic pathways were significantly downregulated upon OA treatment throughout the Cntrl 1, Cntrl 2, and Stea 1 cell lines (Figure 2E, Supplementary Data Sheet S2). Confirming results from a previous study (Graffmann et al., 2021), we found that genes associated with KEGG pathways of peroxisome proliferator activated-receptor (PPAR), adenosine monophosphate-activated protein kinase (AMPK)-signaling, and fatty acid metabolism were significantly upregulated (Figure 2F). Furthermore, we detected genes belonging to inflammation-related pathways that were significantly upregulated upon OA induction, such as tumor necrosis factor (TNF) signaling and NF-kappa-B pathway. Interestingly, genes of the inflammatory bowel disease and insulin resistance pathways were also upregulated upon OA treatment (Figure 2F). This KEGG-associated pathway analysis confirmed the induction of the steatosis phenotype by OA supplementation in the three cell lines Cntrl 1, Cntrl 2, and Stea 1. We performed Pearson’s correlation heatmap analysis of genes associated with relevant KEGG pathways (Figure 3A). Notably, this revealed a change of clustering according to the treatment and independent of the genotype, indicating the relevance of these genes for the phenotype. Taken together, global transcriptomic analyses of Cntrl 1, Cntrl 2, and Stea 1 cell lines confirmed successful steatosis induction but did not show an altered susceptibility of the patient-derived cells.

To confirm the global gene expression changes, we performed qRT-PCR for genes associated with relevant pathways that are depicted in Figure 3B as the relative expression normalized to the mock condition for each cell line. Inter-individual differences are shown in Supplementary Figure S4. We found significant increases in PPAR-pathway associated genes, *PLIN2*, and fatty-acid-binding protein-1 (*FABP1)* upon OA treatment in at least two out of our three cell lines. Regarding insulin signaling-associated genes, phosphoenolpyruvate carboxykinase-1 (*PCK1*) was significantly upregulated in Cntrl 2, and a non-significant trend toward upregulation was detectable in Cntrl 1 and Stea 1. Carnitine palmitoyltransferase-1A (*CPT1A*) was significantly upregulated in Cntrl 1 and 2. Interestingly, in contrast to the RNA-seq results, insulin-like growth factor binding protein-1 (*IGFBP1)* showed a tendency of upregulation upon OA treatment; however, it was not significant. Considering genes associated with inflammation, a significant upregulation of CC-chemokine ligand 4 (*CCL4*) and interleukin 1beta (*IL1B)* expression was detected in Cntrl 1 and Stea 1, whereas in Cntrl 2, the increase was not significant. We found a significant reduction in the expression of vascular cell adhesion molecule-1 (*VCAM1*) in Cntrl 2 and Stea 1. Furthermore, we found members of the renin–angiotensin-system (RAS) differentially regulated in our model. For example, the expression of angiotensin-converting enzyme 2 (*ACE2*) tended to be upregulated in the RNA-seq data, which is a non-significant trend that could be confirmed by qRT-PCR. In addition, we detected a reduction of alanyl aminopeptidase (*ANPEP*), which is also involved in glutathione metabolism (reactive oxygen species (ROS) regulation), in all three cell lines, albeit only significantly in Stea 1. The tendency of up- and downregulation of the qRT-PCR data in Figure 3B confirms the direction of regulation in the RNA-seq data. Together, these findings strengthen the validity of our model because MAFLD-associated pathways were differentially regulated upon OA treatment. They further underline the importance of the investigated genes as their differential expression was independent of the genetic background.

### 3.3 Dipeptidyl peptidase 4 is secreted upon OA treatment

**FIGURE 4:**
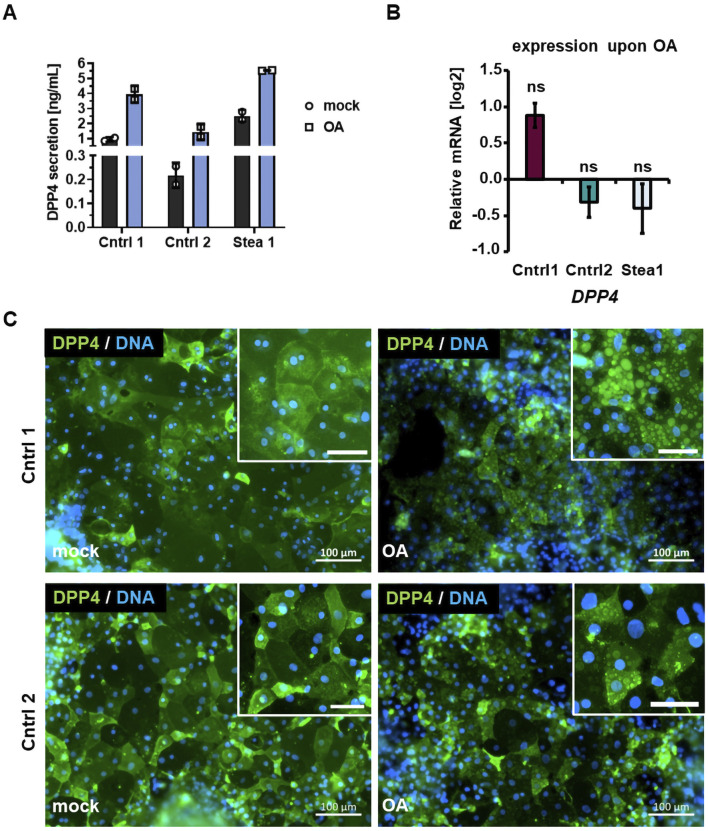
DPP4 is secreted upon OA treatment. **(A)** Secretion of dipeptidyl peptidase (DPP4) upon OA treatment measured by ELISA detection showing the secreted DPP4 in HLCs of two donors as means ±SD. **(B)** Gene expression of *DPP4* upon OA treatment normalized to the housekeeping gene *RPLP0* and relative to the gene expression under the mock condition, shown as mean ± SEM. Two-tailed Student’s T-test was performed to calculate significances (ns = p>0.05). **(C)** Representative immunocytochemistry of DPP4 under mock and OA conditions in cell lines Cntrl 1 and Cntrl 2. Scale bars represent 100 μm and 50 µm in the zoomed-in images.

We analyzed the supernatant of OA-treated HLCs for released signaling proteins. Among others, we found a significant increase of DPP4, also known as cluster of differentiation 26 (CD26), upon OA induction (Supplementary Figure S5A–C). We confirmed this tendency of increase upon OA treatment in Cntrl 1, Cntrl 2, and Stea 1 by ELISA (Figure 4A) (0.95 ± 0.11 ng/mL, 0.22 ± 0.04 ng/mL, and 2.51 ± 0.27 ng/mL DPP4 under mock conditions and 3.94 ± 0.38 ng/mL, 1.45 ± 0.35 ng/mL, and 5.53 ± 0.02 ng/mL DPP4 under OA, respectively).

Interestingly, there was a greater increase in DPP4 secretion upon OA treatment in cell lines derived from healthy individuals compared to patient-derived HLCs. DPP4 levels increased approximately 4.5- to 5-fold in Cntrl 1 and 2, while Stea 1 showed a 2-fold increase in DPP4. This indicates that the control cell lines are able to increase DPP4 secretion more strongly in response to OA. To gain insights into the mechanisms underlying the upregulation of DPP4 upon OA treatment, we analyzed the gene expression in the three cell lines; however, we could not detect a significant change upon OA induction (Figure 4B). Similarly, the closely related proteins DPP8 and 9 were not regulated on the mRNA level after treatment. However, we observed a considerably lower expression of DPP9 compared to that of DPP4 and 8, indicating that it plays a minor role in our system (Supplementary Figure S6). To elucidate the role of DPP4 in steatosis, we focused on the Cntrl lines for further analyses because of the stronger induction of DPP4 secretion upon OA treatment. Similar to the gene expression of *DPP4*, we did not detect a prominent change in the protein localization or amount upon OA treatment (Figure 4C).

### 3.4 Vildagliptin reduces DPP4 activity

**FIGURE 5:**
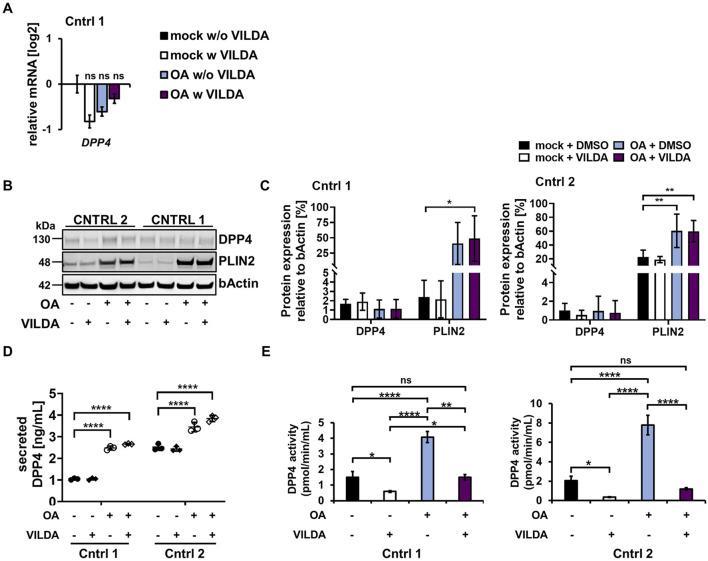
Effects of VILDA on OA-induced HLCs. **(A)** Gene expression of *DPP4* in Cntrl 1 HLCs upon OA treatment with (w) and without (w/o) 30 µM vildagliptin (VILDA) for 5 days in comparison to mock treatment w/o VILDA in HLCs from one donor normalized to the housekeeping gene *RPLP0* and relative to the expression under mock treatment w/o VILDA, shown as means ±SEM. Ordinary one-way ANOVA followed by Tukey’s multiple comparisons was performed to calculate significances (ns p> 0.05). **(B)** Representative cropped WB of DPP4 and PLIN2 upon OA treatment w and w/o VILDA. Uncropped full-length blots are shown in Supplementary Figure S12. **(C)** Protein expression relative to b-actin in comparison to the mock treatment w/o VILDA in Cntrl 1 and 2,shown as means± SD. Ordinary two-way ANOVA, followed by Tukey’s multiple comparison test, was performed to calculate significances (*p <0.05and **p <0.01). Uncropped full-length blots are shown in Supplementary Figure S12. **(D)** DPP4 secretion upon OA treatment w and w/o VILDA, shown as means ±SD. Ordinary two-way ANOVA followed by Dunnett’s multiple comparison test was performed to calculate significances (**p <0.01, ***p <0.001, and ****p <0.001). **(E)** DPP4 activity in HLCs from two donors upon OA treatment w and w/o VILDA was analyzed by measuring the proteolytic activity over time. Ordinary one-way ANOVA and Tukey’s multiple comparison test were performed to calculate significances (*p <0.05and ****p <0.001).

VILDA was tested in a phase-4 study (ID NCT01356381) to elucidate its potential use for treating steatosis patients (Macauley et al., 2015). However, whether VILDA improves the hepatic phenotype directly or by incretin regulation is yet to be elucidated. To shed light on its direct effects on hepatocytes, we induced the steatosis phenotype in our HLCs through pretreatment with OA for 48 h followed by incubation with 30 µM VILDA for a total of 5 days simultaneously with OA. We did not detect significant differences in *DPP4* expression on both the RNA and protein levels upon treatments in comparison to that in mock w/o VILDA (Figures 5A–C), nor did we observe changes in the expression of the DPP4 gene family members DPP8 and 9 (Supplementary Figure S7). However, we detected an expectedly strong increase for PLIN2 after OA induction in Cntrl 1 and 2 HLCs (Figures 5B,C).

Considering the secretion of DPP4, we confirmed the previously detected increase of DPP4 upon OA treatment w and w/o VILDA for both cell lines in comparison to that in the mock treatment. However, no significant difference upon VILDA treatment was detectable (Figure 5D). Nevertheless, we detected a significant increase in DPP4 activity for both cell lines upon OA treatment, which was significantly reduced when HLCs were treated with OA and VILDA together (Figure 5E). VILDA is capable of reducing DPP4 activity to the level detected under mock conditions, with no significant difference between OA w VILDA and mock w/o VILDA. These findings confirm that VILDA mainly acts on DPP4 activity and neither on its gene or protein expression nor on its secretion.

### 3.5 Inhibition of DPP4 activity might reduce inflammatory progression leading to the disease phenotype

To test whether VILDA further affects other genes involved in the steatosis phenotype, we analyzed the global gene expression upon OA w and w/o VILDA in cell line Cntrl 1. To gain the first insights on effects of DPP4 inhibition, we selected Cntrl 1 for in-depth analysis by NGS, which will provide directions for the necessary follow-up studies. The first-level dendrogram analysis revealed clustering according to mock- and OA treatment but not according to the VILDA treatment, indicating a stronger impact of OA on the gene expression in comparison to that of VILDA (Supplementary Figure S8A). To confirm the previously detected gene expression pattern in response to OA, we performed KEGG-associated pathway analysis of the gene expression upon OA treatment w/o VILDA in comparison to mock treatment w/o VILDA (Supplementary Figure S8B,C). Indeed, similar pathways were differentially regulated, indicating that the solvent reagent had no impact on the OA response (Supplementary Figure S8B,C).

**FIGURE 6:**
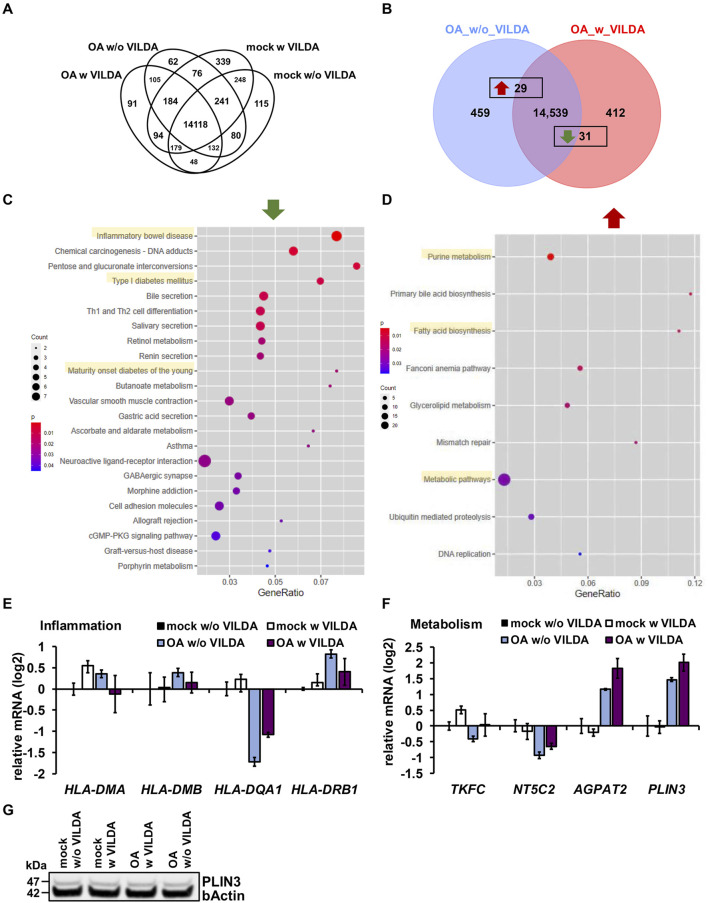
VILDA effects on the global transcription of steatotic Cntrl 1 HLCs. **(A)** Venn diagram of expressed genes upon indicated treatments. **(B)** Venn diagram of the gene expression of OA-treated HLCs w and w/o VILDA. **(C,D)** KEGG-associated pathways of significantly down-**(C)** and up-**(D)** regulated genes upon OA treatment w VILDA (complete gene lists are provided in Supplementary Data Sheet S3). **(E,F)** Expression of inflammation-associated **(E)** and metabolism-associated **(F)** genes upon OA treatment w and w/o VILDA treatment. **(G)** WB of perilipin-3 (PLIN3) upon OA treatment w and w/o VILDA in comparison to housekeeping protein b-actin. Uncropped full-length blots are shown in the Supplementary Figure S12.

In addition to the 14,118 genes expressed in common in all four conditions, we found exclusively expressed gene sets for every condition, as indicated by Venn analysis (Figure 6A). We found 91 and 339 exclusively expressed genes upon OA treatment w VILDA and mock treatment w VILDA, respectively. A total of 62 and 115 genes were exclusively expressed upon OA treatment w/o VILDA and mock treatment w/o VILDA treatment, respectively. KEGG- and GO-associated pathway analyses for the exclusively expressed genes upon the different conditions did not reveal characteristic profiles (Supplementary Data Sheet S3), except for an exclusive expression of genes associated to the KEGG pathways that are related to cancer after VILDA treatment (Supplementary Data Sheet S3). In general, our findings demonstrate a mild effect of VILDA compared to that of OA, and for further analyses, we included both the exclusively and common but differentially expressed genes.

Next, we wanted to know whether VILDA influences gene expression upon OA treatment and found 29 genes significantly up- and 31 genes significantly downregulated upon VILDA treatment (Figure 6B). KEGG pathway analysis revealed that genes belonging to the inflammatory bowel disease pathway, asthma, and type 1 diabetes mellitus (T1DM)-associated pathways were significantly downregulated (Figure 6C;Supplementary Figure S10). Common downregulated genes of these pathways encode members of the human leukocyte antigen II (HLAII) family, namely, HLA-DQA1 and HLA-DMB, which are known to be ectopically expressed on hepatocytes upon hepatitis (Lu et al., 2020a; Lu et al., 2020b; Dezhbord et al., 2024). Although the expression changes were small, we confirmed upregulation of *HLA-DMA*, *HLA-DMB*, and *HLA-DRB1* upon OA treatment and a tendency toward downregulation upon OA treatment w VILDA (Figure 6E). Interestingly, in contrast to the RNA-seq results, we found *HLA-DQA1* expression downregulated upon OA treatment and a slight increase of expression upon OA w VILDA treatment (Figure 6E). These data might indicate a potential association between DPP4 and HLAs in steatosis. However, experiments with more cell lines and prolonged treatment would be necessary for confirmation.

Genes associated with metabolic pathways, such as purine metabolism and fatty acid biosynthesis pathways, were upregulated in OA w VILDA versus OA w/o VILDA treatments (Figure 6D; Supplementary Figure S10). Interestingly, we found triokinase and FMN cyclase (*TKFC*) and 5′-nucleotidase, cytosolic II (*NT5C2*), both members of purine metabolism, downregulated upon OA treatment w/o VILDA in comparison to mock treatment w/o VILDA, whereas VILDA treatment induced an upregulation, which might indicate the restoration of the pathway (Figure 6F). Considering fatty acid metabolism, we detected that O-acyltransferase 2 (*AGPAT2*) was upregulated upon OA w/o VILDA treatment in comparison to mock treatment w/o VILDA, which was even reinforced upon OA treatment w VILDA (Figure 6F). We saw the same trend in the gene expression of perilipin-3 (*PLIN3*); however, we did not detect differential protein expression (Figure 6F,G). In contrast, considering another memeber of the perilipin family, namely PLIN2, we detected the same gene expression pattern, but differential protein expression. (Figure 6F, Supplementary Figure S9B). PLIN2 protein levels were upregulated upon OA treatment, while PLIN3 protein levels were stable throughout the conditions. A possible reason is that PLIN3 belongs to the exchangeable PLINs, while PLIN2 is a constitutive protein, which is upregulated upon OA treatment and unstable in the absence of LDs (Wolins et al., 2006). PLIN3 is stable in the cytoplasm independently of LDs but is recruited to the lipid fractions (Wolins et al., 2006). Nevertheless, considering the mRNA expression, our findings might indicate that DPP4 inhibition upon OA treatment restores the hampered cellular energy homeostasis; however, further studies are necessary to pinpoint the underlying mechanism.

To test whether VILDA affects the expression of steatosis-associated genes, we analyzed the expression of genes belonging to the PPAR and gluconeogenesis pathways, which were both relevant in our previous studies, via heatmaps and detected clustering according to each condition (Supplementary Figure S13, S14). In addition, we evaluated the steatosis gene set from the earlier global analysis. We could confirm the same trend of gene expression for Cntrl 1 upon OA treatment (Supplementary Figure S9A), but we could not detect a significant difference when comparing OA treatment w/o and w VILDA.

To gain insights into the overall effect of VILDA, independent of the steatosis condition, we performed KEGG pathway analysis of the genes differentially expressed under mock conditions. We found 47 genes that were significantly upregulated and 44 genes that were significantly downregulated upon mock treatment w and w/o VILDA (Supplementary Figure S11A). KEGG-associated pathway analysis revealed, among others, downregulated genes associated with inflammatory response pathways under mock conditions (Supplementary Figure S11B). Upregulated genes were involved in KEGG-associated pathways such as enhanced metabolism and insulin secretion, indicating beneficial effects for the compromised insulin pathway, since the insulin resistance pathway was upregulated upon OA treatment (Supplementary Figure S11C). Altogether, our findings support the hypothesis that DPP4 is involved not only in metabolic regulation via purine and fatty acid metabolism but furthermore, it also affects inflammatory-associated pathways. It should be noted that we cannot exclude a potential additional inhibitory activity of VILDA on DPP8/9 caused by the structural homology of the three proteases (Burkey et al., 2008). Indeed, we detected low levels of DPP9 expression and higher DPP8 expression in our steatotic hepatocytes (Supplementary Figures S6, S7) after normalization.

## 4 Discussion

### 4.1 Oleic acid feeding induces the clinically relevant phenotype of steatosis

In this study, we generated iPSC-derived steatotic HLCs from four individuals to elucidate the potential hepatocyte-specific contribution to the progression of MAFLD. We and others have shown that iPSC-derived HLCs with considerable metabolic activity are a valuable system to model MAFLD, reflecting the diverse genetic backgrounds of the donors (Wruck et al., 2015; Graffmann et al., 2016; Wruck et al., 2017; Yu et al., 2024; Xiong et al., 2024). Steatosis can be induced by treating the cells with glucose, pyruvate, or lactate; however, the most common method is the combination of saturated and unsaturated fatty acids (Ramos et al., 2022). To induce hepatic steatosis for a relatively long period of 7 days, we used an unsaturated fatty acid as they are considered to be less apoptotic and less damaging for the cells (Ricchi et al., 2009). By stimulating HLCs with OA, we were able to induce lipid droplet formation in all four cell lines, as already shown in previous studies (Wruck et al., 2015; Graffmann et al., 2016). Although two of the cell lines were derived from male and female steatosis patients and healthy individuals, respectively, there was no detectable disease-specific effect or sex-dependent effect w or w/o OA. Interestingly though, our previous findings indeed indicated steatosis related gene expression patterns (Graffmann et al., 2021). Nevertheless, cell line-specific differences in the amount and size of the lipid droplets were observed. This is in line with our previously published data and the high divergence in individual symptoms and progression of MAFLD, which are well-known difficulties in clinical practice (Wruck et al., 2015). They are due to genetic variations like single nucleotide polymorphism (SNPs), epigenetic alterations and other co-morbidities that are associated with the disease (Yu et al., 2024; Miyazak et al., 2012). Furthermore, considerable variability is typical for iPSC-derived data due to the individual genetic background and differences in differentiation efficiencies. Indeed, we excluded the patient-derived cell line Stea 2 due to ambiguous clustering in the first-level analysis. In addition, sex-dependent effects are recognized driving factors in the MAFLD pathology, with men and post-menopausal women being more susceptible to the disease than young women (Balakrishnan et al., 2021). However, our small cohort of two female and two male samples precludes any conclusions regarding sex-related effects because of the aforementioned multifactorial characteristic of the disease (Grzych et al., 2020; Abdelnabi et al., 2022). A much larger sample set-up is warranted to prevent misinterpretation caused by inter-individual differences.

Global transcriptomic analyses revealed that MAFLD-associated pathways such as metabolism-associated and immune-modulating pathways were differentially regulated in our model (Wruck et al., 2017; Yu et al., 2024; Xiong et al., 2024). A heatmap analysis of genes from these pathways revealed clustering according to the treatment and independent of the genetic background. This underlines the importance of the involved genes for the disease. Although our model comprises only hepatocytes, the detected upregulation of cytokine–cytokine receptor signaling, NF-kappa B, and TNF signaling upon OA treatment might indicate immune cell recruitment via inflammatory/chemokine signaling. Recently, Yu et al. demonstrated that hepatocyte-intrinsic changes contribute to the disease (Yu et al.,2024), while predisposition, environment, or other comorbidities might further regulate the pace of the progression toward fibrosis and cirrhosis. Comparing the gene expression profile detected in our HLCs with their single-cell RNA-seq data from liver resections of NASH and HCC patients, we found many upregulated pathways that are associated with a lower risk of NASH–HCC transition, such as galactose catabolic processes, hexose metabolic processes, and glucose homeostasis.

### 4.2 Active dipeptidyl peptidase 4 is secreted upon OA treatment

DPP4 is a serine protease with catalytic activity for various substrates (Mulvihill and Drucker, 2014) and considered a hepatokine, upregulated in metabolic liver disease and driver of inflammation (Trzaskalski et al., 2020. Similar to observations made in HepG2 cells (Miyazak et al., 2012), we could not detect elevated DPP4 on both the mRNA or protein levels. However, we found elevated secretion and a drastic increase in the catalytic activity of DPP4 upon OA treatment, which is in line with a previously observed increase in NAFLD/NASH patients (Barchetta et al., 2021; Barchetta et al., 2022). Since DPP4 is involved in various chronic and cancerous diseases throughout the human body, hepatocyte-specific secretion upon late steatosis might indicate inflammatory signaling and a risk for the development of other comorbidities such as cardiovascular and renal diseases (Tomovic et al., 2019).

### 4.3 VILDA interferes with DPP4 activity and regulates pathways related to inflammation and metabolism

As a first insight into the mechanism of action of DPP4, we inhibited its activity with VILDA—an FDA-approved T2DM medication. Although VILDA can also have an effect on the activity of other family members such as DPP8/9 and FAP, the effects observed in this study are most likely driven by DPP4 since our cells expressed DPP9 only at a low level and FAP was not expressed at all (Supplementary Figures S6, S7). Nevertheless, it is notable that as DPP9 is involved in immune regulation, future studies focusing on its potential role in steatosis are necessary (Waumans et al., 2015; Cui et al., 2022). As a proof-of-principle, we found that VILDA interferes with DPP4 activity and not with its protein or mRNA levels, as described in previous studies (Mathieu and Degrande, 2008). In our study, as the first in this format, global transcriptomic analysis revealed clustering according to the OA/mock treatment but not according to VILDA treatment. This confirmed our expectation that OA treatment induced greater transcriptomic changes than VILDA treatment. Nevertheless, we detected exclusively expressed genes for all four conditions. Although KEGG-associated pathway analysis did not identify any characteristic profiles, we noticed that genes involved in the development of cancer were upregulated upon VILDA treatment independently of steatosis. VILDA-associated safety concerns have already been addressed extensively, and no significant overall cancer-association was found (Zhao et al., 2017; Kanazawa et al., 2017). Considering the typical long-term or even life-long medication of T2DM, this should, nevertheless, be monitored carefully.

Furthermore, we found inflammation-associated pathways such as T1DM, inflammatory bowel disease, and asthma downregulated upon DPP4 inhibition. *HLA-DQA1* and *HLA-DMB* are common genes involved in all of them, and their expression was differentially regulated upon steatosis and additional DPP4 inhibition. HLA class-II proteins are typically expressed on the surface of antigen-presenting cells; however, ectopic expression in hepatocytes upon disease has been shown (Lu et al., 2020a; Dezhbord et al., 2024). In line with this, we found a tendency of elevated gene expression of *HLA-DMB*, *HLA-DMA*, and *HLA-DRB1* upon OA treatment, which are associated with NASH, hepatitis, and cirrhosis (Zhang et al., 2023; Lu et al., 2014), and they all decreased upon DPP4 inhibition. Since DPP4 activity affects (auto)-immune related diseases in a complex manner (Huang et al., 2022), this might provide a possible clue regarding its role in steatosis/MAFLD progression. However, further studies are necessary to elucidate the role of DPP4 in the steatosis model and to decipher whether partial effects were caused because of potential DPP8/9 inhibition by VILDA.

To understand the role of DPP4 in the interplay between metabolism and inflammation, it is essential to determine whether DPP4 is causal or correlative for late steatosis. DPP4 was shown to be epigenetically regulated (Hyun and Jung, 2020; Saussenthaler et al., 2019). Indeed, we also found a slight, albeit not significant, demethylation upon OA treatment (not shown). This supports the speculation that early events of energy overload might change the methylation profiles of CpG islands in the DPP4 locus and enable DPP4 expression in hepatocytes at a rather early time-point of disease progression.

The insulin resistance pathway was upregulated upon OA treatment, which matches with the well-known insulin resistance-promoting effect of DPP4. In addition, genes involved in PPAR signaling and gluconeogenesis showed condition-dependent gene expression patterns. Interestingly, we found genes involved in purine and fatty acid metabolism upregulated upon VILDA treatment. E.g. *AGPAT2*, which is involved in fatty acid metabolism, was upregulated upon DPP4 inhibition. Its deletion or mutation is associated with insulin resistance, diabetes, and severe forms of metabolic syndrome in mice and humans (Cortés et al., 2009; Araújo-Vilar and Santini, 2019; Agarwal et al., 2024). This could indicate a VILDA-mediated beneficial effect for hampered metabolism due to the energy overload during late steatosis and support the idea of a role of DPP4 in the interplay between metabolism and inflammation (Ghorpade et al., 2018; Baumeier et al., 2017).

## 5 Conclusions and outlook

Taken together, we provide a human iPSC-derived model focusing on the hepatocyte-specific contribution to progression of steatosis. We could link DPP4 activity to the steatosis phenotype and show that its inhibition with VILDA has effects on metabolism- and inflammation-associated gene expression during steatosis. Since we performed global transcriptomic analyses of the effects of VILDA with only one cell line, these can only provide first insights into possible effects, and more in-depth analyses are needed. In the future, human DPP4 knockout HLCs, embedded in a multicellular liver model, could increase our understanding of the mechanisms of DPP4 through health and disease and help in further elucidating the interplay between the distinct cell types of the liver.

## Data Availability

The data presented in the study are deposited in the GEO repository, accession number GSE310214 and GSE310216.

## Glossary

ACE2: Angiotensin-converting enzyme 2
AFP: Alpha-fetoprotein
AGPAT2: O-acyltransferase 2
ALB: Albumin
AMPK: Adenosine monophosphate-activated protein kinase
ANPEP: Alanyl aminopeptidase
CCL4: C-C motif chemokine ligand 4
CD26: Cluster of differentiation 26
Cntrl: Control
CPT1A: Carnitine palmitoyltransferase I
CYP: Cytochrome P450
DE: Definitive endoderm
Dex: Dexamethasone
DPP4: Dipeptidyl peptidase 4
E-CAD: E Cadherin
ELISA: Enzyme-linked immunosorbent assay
FABP1: Fatty acid-binding protein 1
FDA: Food and Drug Administration (U.S.)
GOs: Gene Ontologies
GSK-3: Glycogen synthase kinase 3
HE: Hepatic endoderm
HGF: Hepatocyte growth factor
HLAII: Human leukocyte antigen II
HLCs: Hepatocyte-like cells
HNF4a: Hepatocyte nuclear factor 4 alpha
ICC: Immunocytochemistry
IGFBP1: Insulin-like growth factor-binding protein 1
igG: Immunoglobulin G
IL1B: Interleukin 1 beta
iPSCs: Induced pluripotent stem cells
KEGG: Kyoto Encyclopedia of Genes and Genomes
LD: Lipid droplet
MAFLD: Metabolic dysfunction-associated fatty liver disease
MASLD: Metabolic dysfunction-associated steatotic liver disease
NAFLD: Non-alcoholic fatty liver disease
NASH: Non-alcoholic steatohepatitis
NT5C2: 5′-nucleotidasecytosolic II
OA: Oleic acid
OCT4: Octamer-binding transcription factor 4
OSM: Oncostatin M
P/S: Penicillin/streptomycin
PCK1: Phosphoenolpyruvate carboxykinase 1
PLIN2: Perilipin-2
PLIN3: Perilipin-3
PPAR: Peroxisome proliferator activated-receptor
qRT-PCR: Quantitative reverse transcription PCR
R.L.U.: Relative light units
RNA-seq: RNA sequencing
ROS: Reactive oxygen species
RPLP0: Ribosomal protein lateral stalk subunit P0
SNPs: Single-nucleotide polymorphisms
SOX: SRY-related HMG-box genes
Stea: Steatosis
T1DM: Type 1 diabetes mellitus
T2DM: Type 2 diabetes mellitus
TBS-T: Tris-buffered saline with Tween20
TKFC: Triokinase and FMN cyclase
TNF: Tumor necrosis factor
VCAM1: Vascular cell adhesion molecule 1
VILDA: Vildagliptin
