# Supplementary material for: DPP4 inhibition affects metabolism and inflammation associated pathways in hiPSC-derived steatotic HLCs
Source: Front Cell Dev Biol. 2026 Feb 26;14:1686556. doi: 10.3389/fcell.2026.1686556 (PMC12979448; doi:10.3389/fcell.2026.1686556)
Supplement: Supplementary file 2 [file DataSheet1.pdf]

Supplementary Material

Table of content

1 Supplementary Methods 2
1.1 Next generation sequencing and analysis of deep sequencing data 2
2 Microscopic imaging data 2
3 Supplementary Figures 2
Fig. S1 Cell morphology during the differentiation stages of cell lines derived from four donors 3
Fig. S2 Characterization of HLC differentiation. (A-C) 4
Fig. S3 OA-induction of Lipid droplets 6
Fig. S4 Gene expression of steatosis markers, inter-individual differences 7
Fig. S5 Released proteins after OA treatment 8
Fig. S6 Euclidean distance of gene expression of DPP family members in HLCs 9
Fig. S7 Euclidean correlation of gene expression of DPP family members in HLCs 10
Fig. S8 Global transcriptome analysis of HLCs treated with OA with and without VILDA 11
Fig. S9 Gene expression of steatosis markers in HLCs treated with OA w and w/o VILDA 12
Fig. S10 Pearson's correlation heatmap analysis upon OA w and w/o VILDA 13
Fig. S11 Global transcriptome analysis of mock-treated HLCs with and without VILDA 14
Fig. S12 Uncropped full-length western blot membranes 15
Fig. S13 Person's correlation heatmap analysis of genes of the gluconeogenesis pathway. 17
Fig. S14 Person's correlation heatmap analysis of genes of the PPAR signaling pathway 18
4 Supplementary Tables 19
4.1 Table S1: List of Primers 19
4.2 Table S2: List of Antibodies 21
6 References 23

1 Supplementary Methods

1.1 Next generation sequencing and analysis of deep sequencing data

The HISAT2 (version 2.1.0) software(1) was employed to align the fastq files received from the BMFZ core facility against the GRCh38 genome. Parameter optimizations(2) were applied resulting in the command:

hisat2 -p 7 -N 1 -L 20 -i S,1,0.5 -D 25 -R 5 --mp 1,0 --sp 3,0 -x hisatindex/grch38_r109 -U input.fastq.gz -S output.sam

Via SAMtools software(3) the resulting BAM files were sorted by coordinates. Read counts per gene obtained with the subread (1.6.1) featurecounts software(4) using the ENSEMBL annotation file Homo_sapiens.GRCh38.109.gtf and parameters –t exon –g gene_id. Within the R/Bioconductor environment data was normalized with the voom(5) algorithm from the limma package(6) filtering genes, which were expressed with CPM (counts per million) > 1 in at least one sample. Venn diagrams were drawn with the VennDiagram package(7) based on genes considered expressed when there were more than 5 reads. Differential expression was determined by a p-value < 0.05 from the limma test and a fold change greater than 1.5 for genes expressed at least in one condition. The False-Discovery-Rate (FDR) was calculated by the method of Storey et al. implemented in the Bioconductor package qvalue (8) The complete correlation table and gene lists for OA/mock experiments can be found in the supplementary table 1. The complete correlation table and gene lists for OA w and w/o VILDA experiments can be found in the supplementary data sheet S2, S3.

2 Microscopic imaging data

Make and model of microscope: Zeiss, LSM 700 microscope

Type, magnification, and numerical aperture of the objective lenses: LD Plan-Neofluar 20x/0.4 and Plan-Apochromat 40x/1.4 Oil DIC (UV)VIS-IR

Temperature: 20-22 °C

Imaging medium: Phosphate-buffered saline (PBS) w/o magnesium and calcium, 1x Fluoromount-G W DAPI (Biozol, Cat. Number: SBA-0100-20)

Fluorochromes: Alexa Fluor 488 Dye, Alexa Fluor 594 Dye, Alexa Fluor 555 Dye, Alexa Fluor 647 Dye, Hoechst 33342

Camera make and model: Zeiss, AxioCam MRM

Acquisition software: Zeiss, ZEN2012 (blue edition), Version 6.1.7601 for fluorescence microscopy and ZEN2011 SP3 (black edition), Version 8,1,6,484 for confocal microscopy. Images were processed using ZEN software, Version 3.10.103.00000,

3 Supplementary Figures

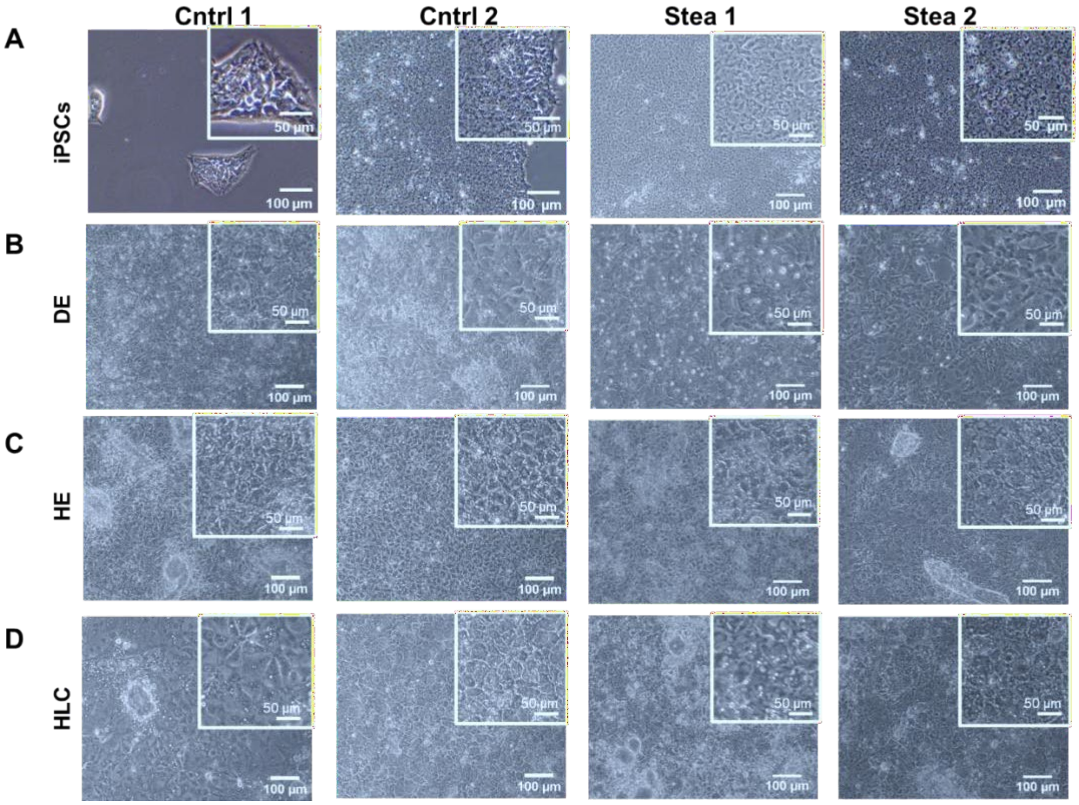

Fig. S1 Cell morphology during the differentiation stages of cell lines derived from four donors . Scale bars represent 100 µm and 50 µm in the zoom-in. **(A)** induced pluripotent stem cells (iPSCs). **(B)** Definitive endoderm (DE). **(C)** Hepatic endoderm (HE). **(D)** Hepatocyte-like cells (HLCs).

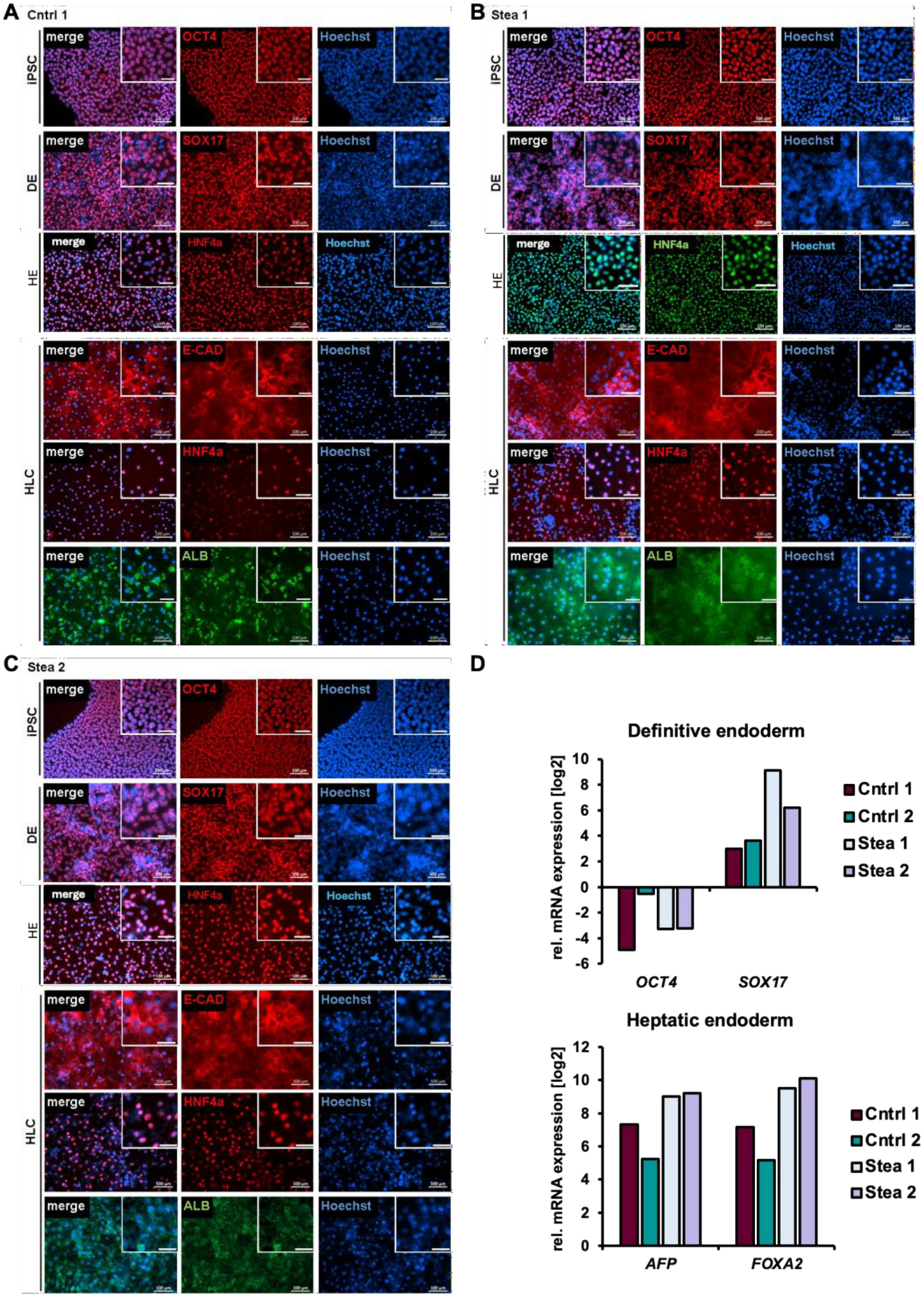

Fig. S2 Characterization of HLC differentiation. (A-C) Representative immunocytochemistry of cell line Cntrl 1, Stea 1 and Stea 2 of differentiation stages showing respective markers OCT4 in

induced pluripotent stem cells (iPSCs), SOX17 in Definitive Endoderm (DE) and HN4a in Hepatic Endoderm (HE). For hepatocyte-like cells (HLCs), the epithelial marker E-CAD, HNF4a and ALB are shown. **(D)** Gene expression of HLCs derived from four cell lines. Shown are means from one preparation analyzed in three technical replicates of *OCT4* and *SOX17* in DE and *AFP* and *FOXA2* in HE in comparison to iPSC-stage.

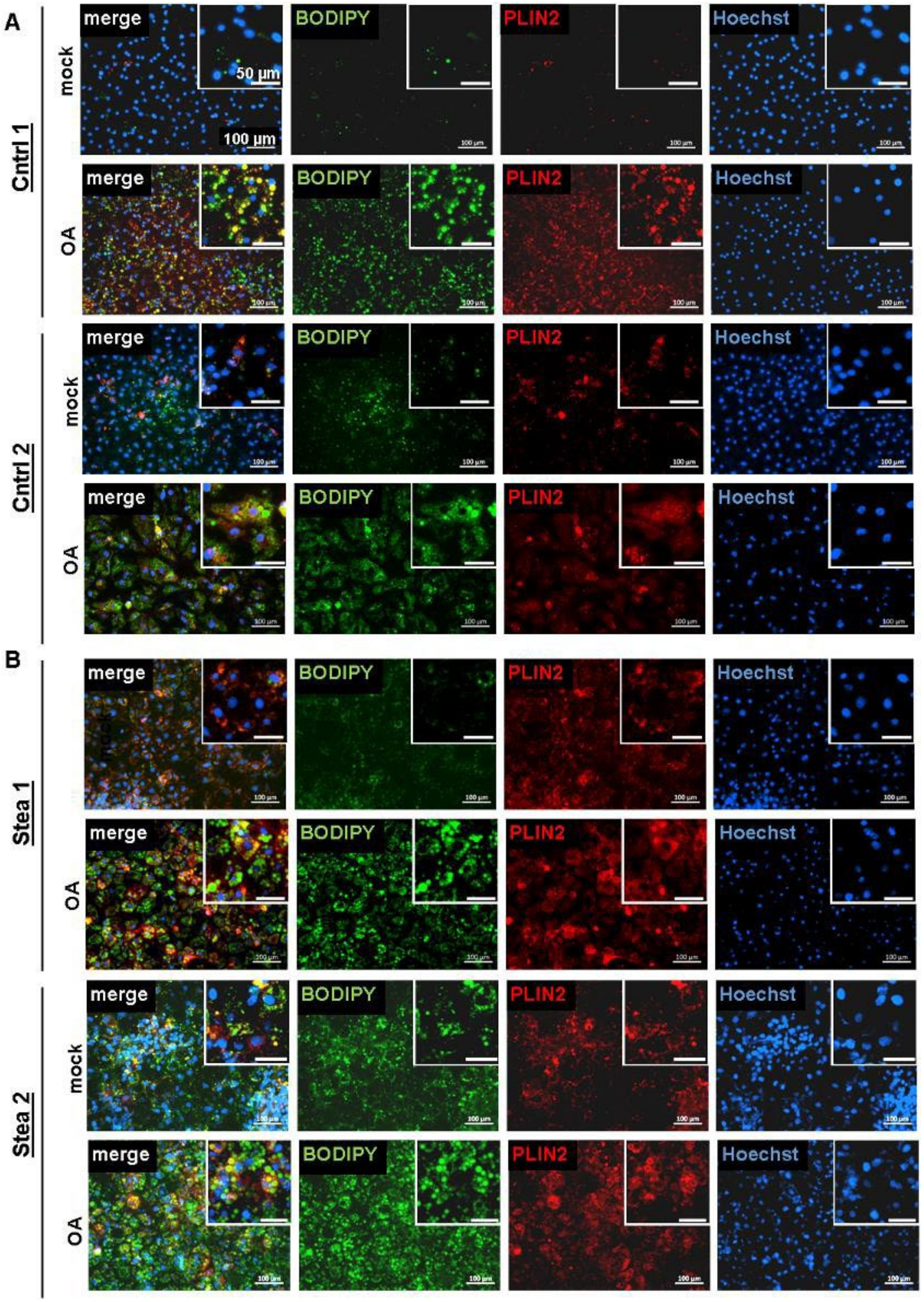

Fig. S3 OA-induction of Lipid droplets. (A/B) Representative immunofluorescence and BODIPY493/503 staining of HLCs of Cntrl 1, Cntrl 2 Stea 1 and Stea 2 HLCs treated with 400 µM OA (OA) and respective control (mock) for 7 days. PLIN2 is shown in red, fatty acids are stained in green, scale bars represent 100 µm in the original image and 50 µm in the zoom-in.

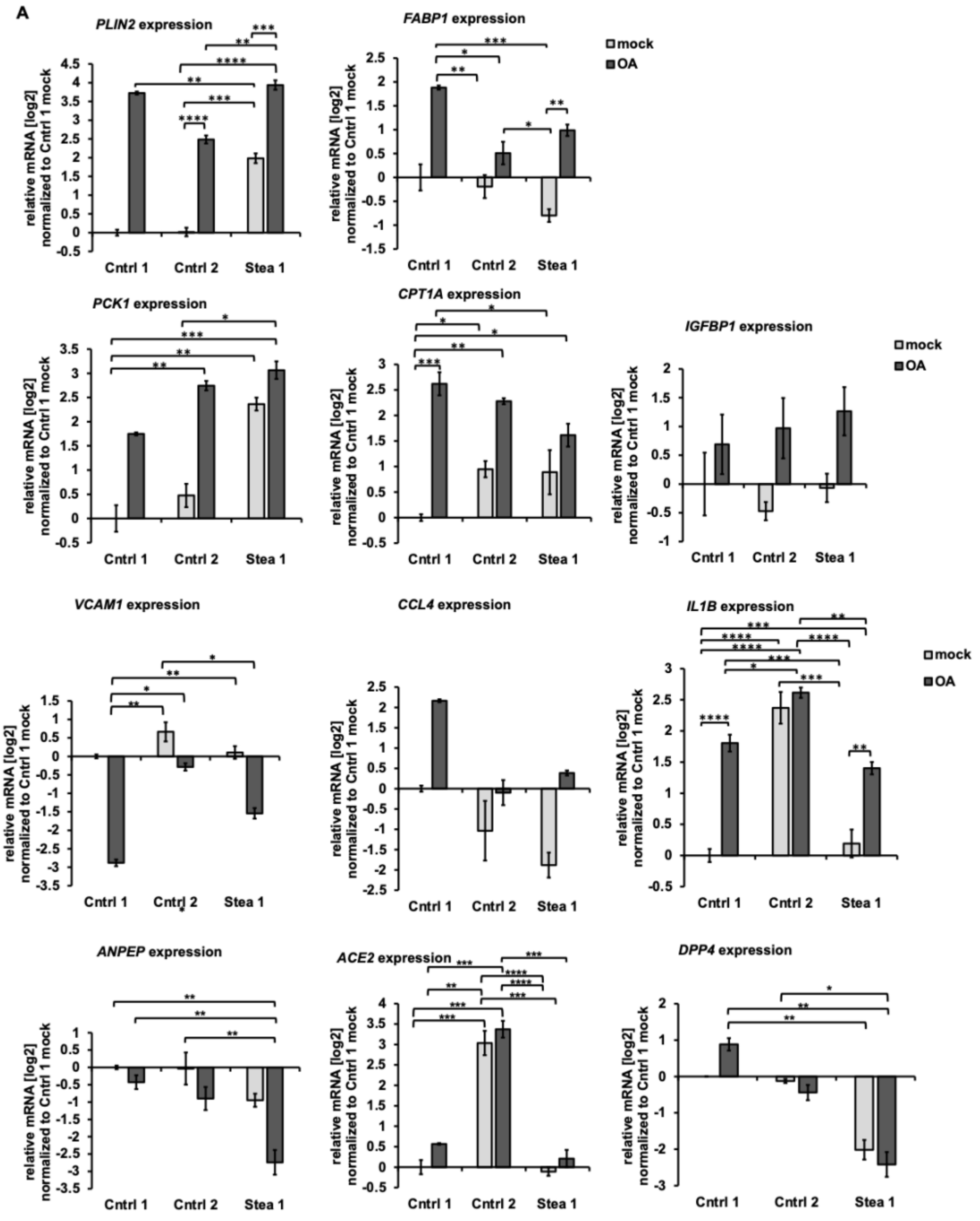

Fig. S4 Gene expression of steatosis markers, inter-individual differences.
Gene expression of PLIN2, FABP1, PCK1, CPT1A, IGFBP1, CCL4, IL1B, VCAM1, ACE2, ANPEP and DPP4 in HLCs from three donors, shown as means ±SEM, normalized to mock w/o VILDA. Ordinary one-way ANOVA, followed by Tukey`s multiple comparison test was performed to calculate significances (*p < 0.05, **p < 0.01, ***p < 0.001 in comparison to Cntrl 1 mock).

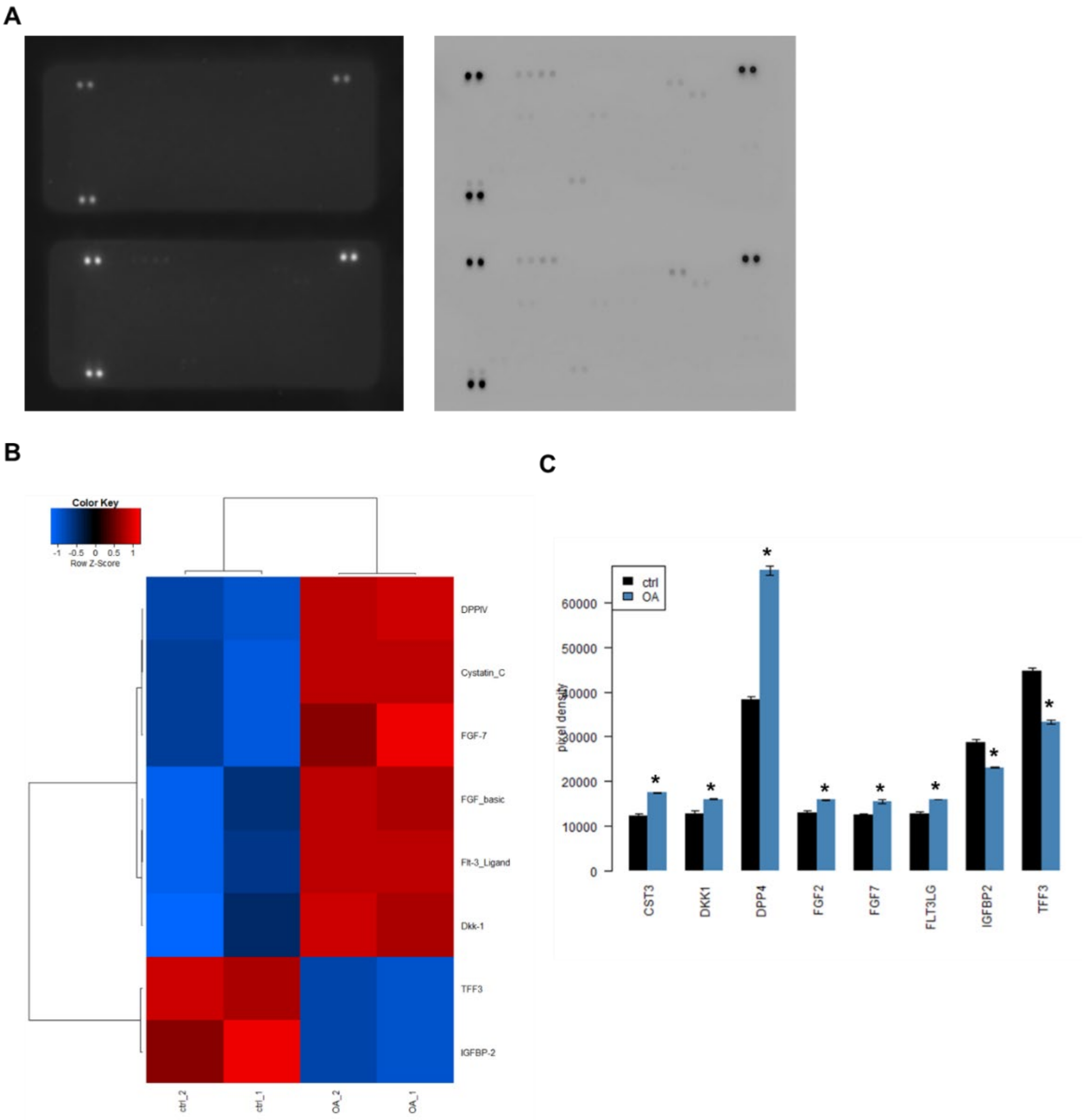

Fig. S5 Released proteins after OA treatment. Cntrl 1 HLCs were treated with OA for 7 days and the supernatant was analyzed for secreted proteins. **(A)** Array membranes, incubated with pooled supernatant of three biological replicates from mock (upper)- or OA (lower) treated Cntrl 1 HLCs. **(B)** Heatmap indicating significantly regulated proteins after analysis of the captured proteins on the membranes in technical duplicates. **(C)** Histogram of the detected chemiluminescence signal in pixel density for captured proteins under mock (ctrl) and OA treatment (* p-value < 0.05).

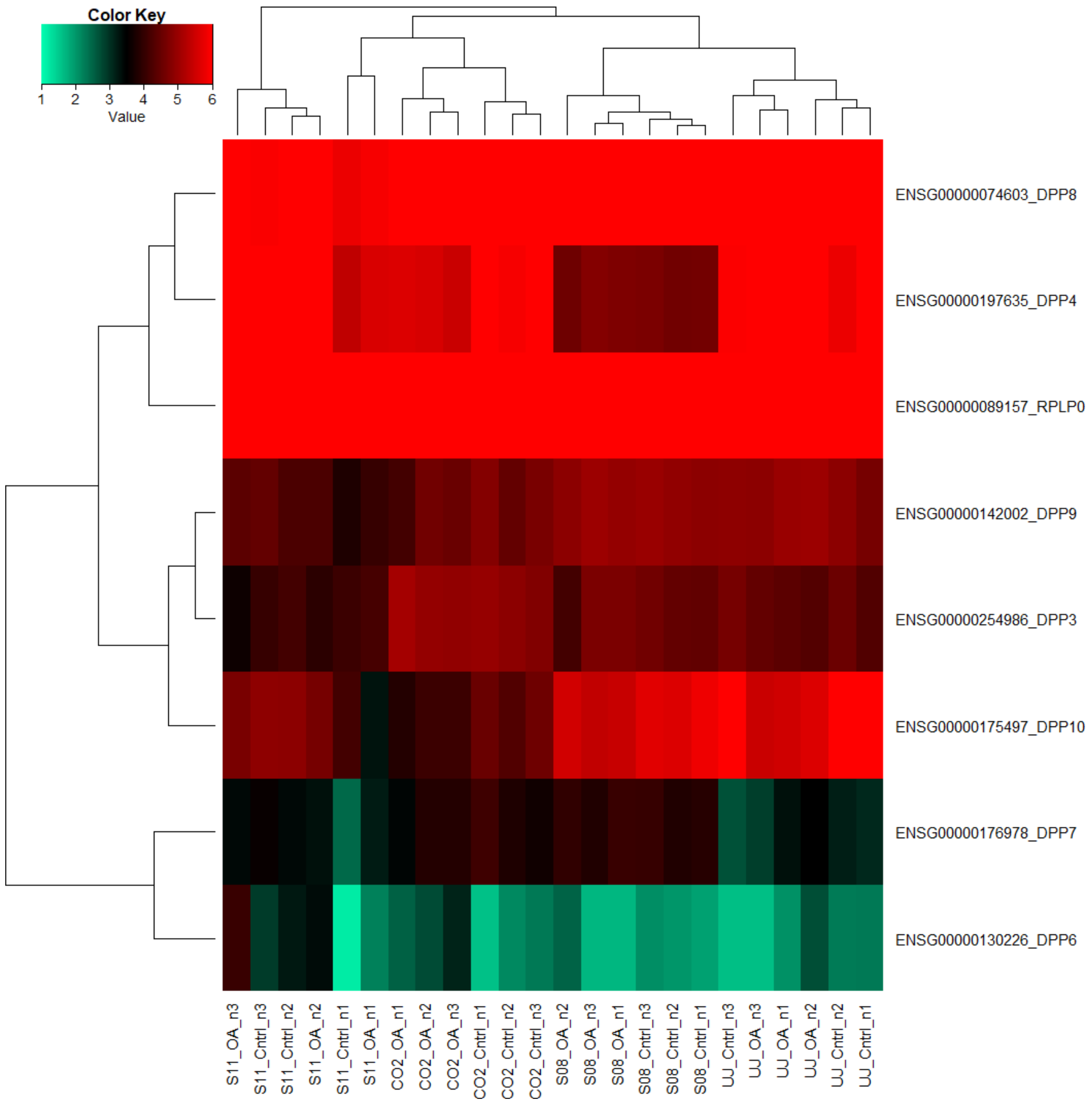

Fig. S6 Euclidean distance of gene expression of DPP family members in HLCs. Euclidean distance heatmap of HLCs from three donors treated with OA or mock, indicating strong gene expression in red while low gene expression is indicated in green. Cntrl = mock treatment, UJ = Cntrl 1, CO2 = Cntrl 2, S08 = Stea1, S11= Stea 2. To allow relative detection of gene expression, expression of the housekeeping gene *RPLP0* is included.

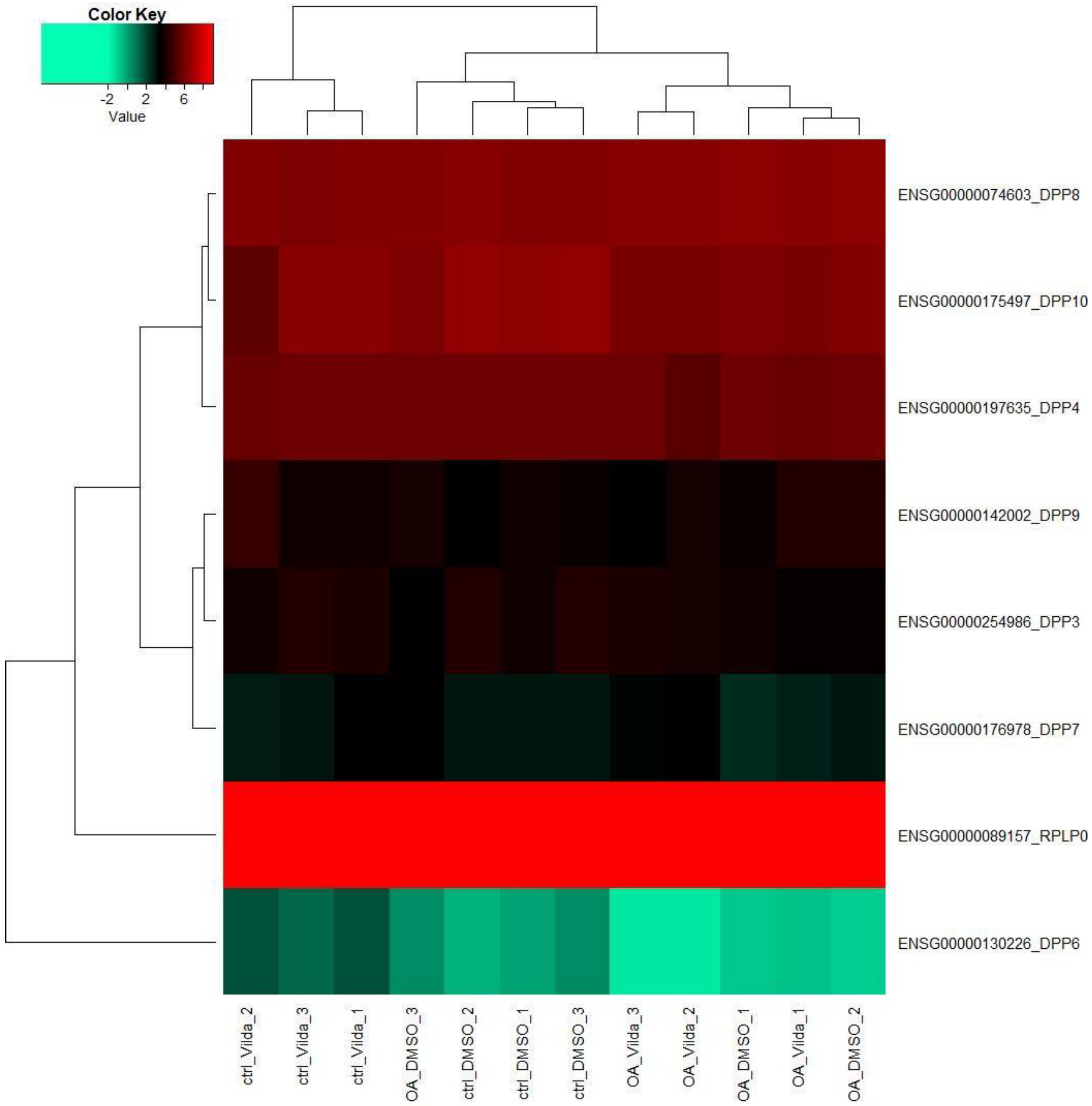

Fig. S7 Euclidean distance of gene expression of DPP family members in HLCs. Euclidean distance heatmap of Cntrl 1 HLCs treated with OA w and w/o VILDA, indicating strong gene expression in red while low gene expression is indicated in green. Ctrl = mock treatment, DMSO = w/o VILDA (solvent control). To allow relative detection of gene expression, expression of the housekeeping gene *RPLP0* is included.

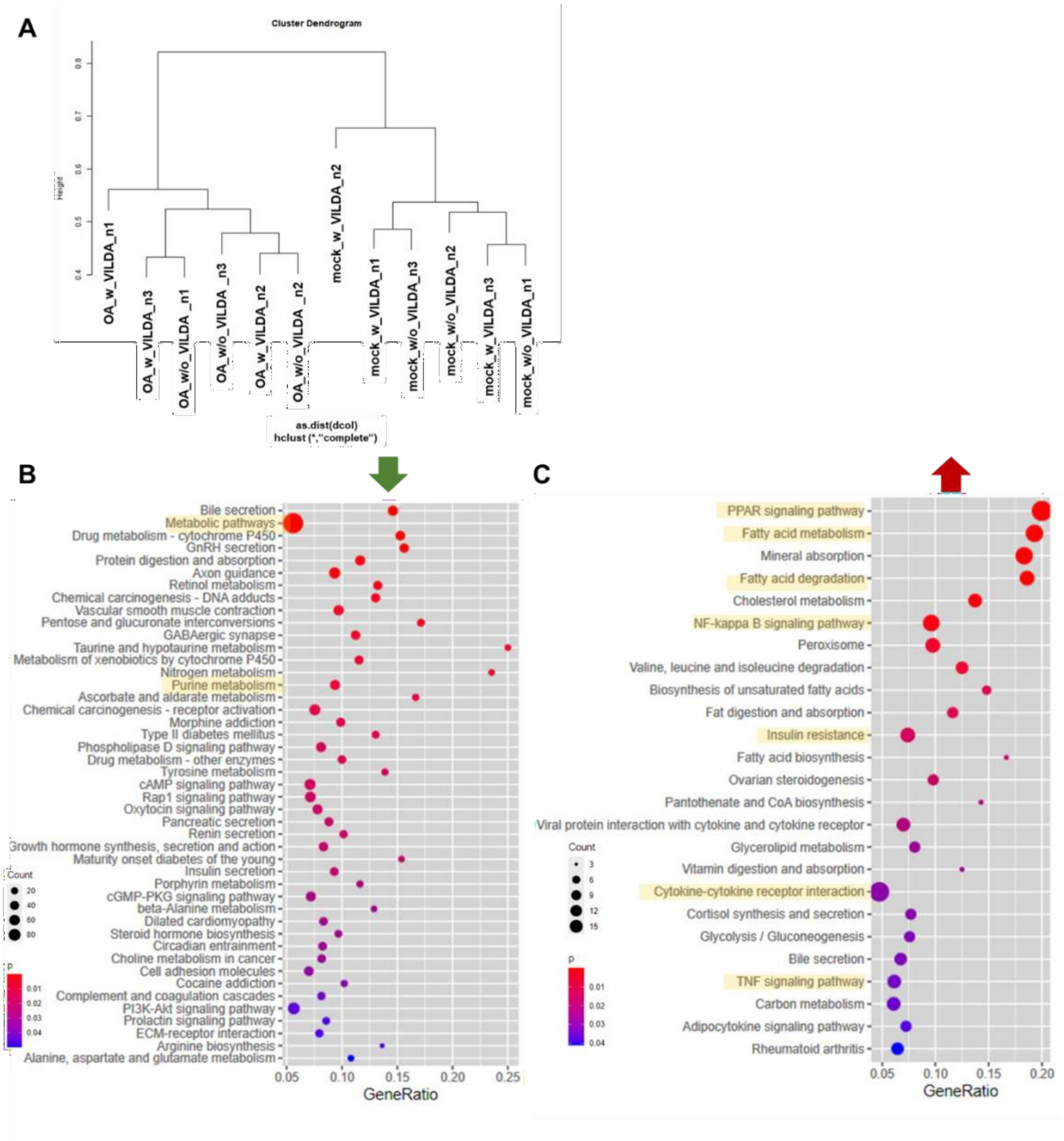

Fig. S8 Global transcriptome analysis of HLCs treated with OA with and without VILDA. (A) Hierarchical cluster dendrogram of global transcriptomic changes upon OA (mock or OA) with and without VILDA (w or w/o VILDA) treatment of HLCs derived from cell line Cntrl 1. **(B)** KEGG-associated pathway analysis of significantly downregulated genes upon OA w/o VILDA in comparison to mock w/o VILDA treatment. **(C)** KEGG-associated pathway analysis of significantly upregulated genes upon OA w/o VILDA in comparison to mock w/o VILDA treatment.

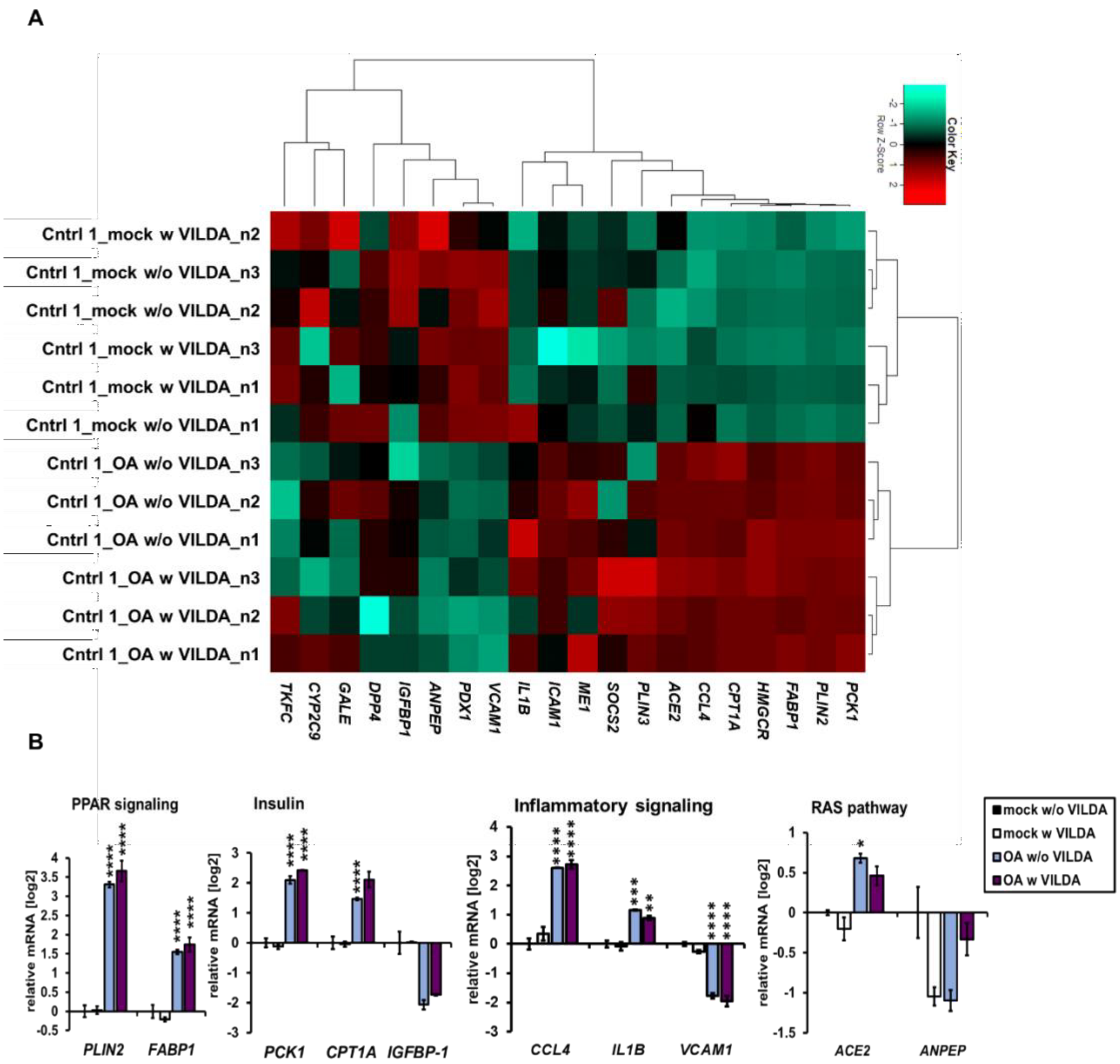

Fig. S9 Gene expression of steatosis markers in HLCs treated with OA w and w/o VILDA. (A) Heatmap analysis of steatosis associated genes. **(B)** Gene expression of *PLIN2*, *FABP1*, *PCK1*, *CPT1A*, *IGFBP1*, *CCL4*, *IL1B*, *VCAM1, ACE2*and *ANPEP*, in HLCs from cell line Cntrl 1 shown as means±SEM, normalized to mock w/o VILDA. Ordinary two-way ANOVA, followed by Tukey`s multiple comparison test was performed to calculate significances (*p < 0.05, **p < 0.01, ***p < 0.001 in comparison to mock w/o VILDA).

A

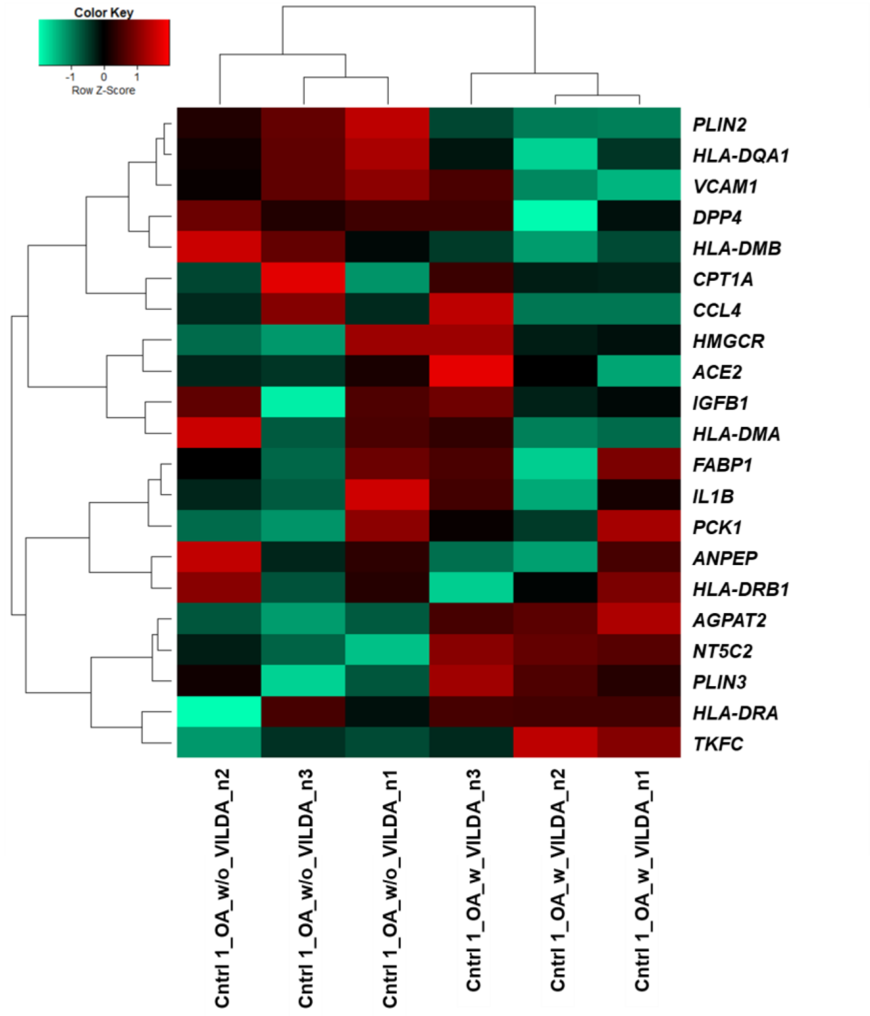

Fig. S10 Pearson's correlation heatmap analysis upon OA w and w/o VILDA. (A) Pearson's correlation heatmap analysis of genes involved in KEGG-associated pathways of purine metabolism (*TKFC*, *NT5C2*), fatty acid metabolism (*FABP1*, *PLIN3*, *AGPAT2*), inflammatory bowel disease, asthma and maturity onset diabetes of the young (*HLA-DQA1*, *HLA-DMB*).

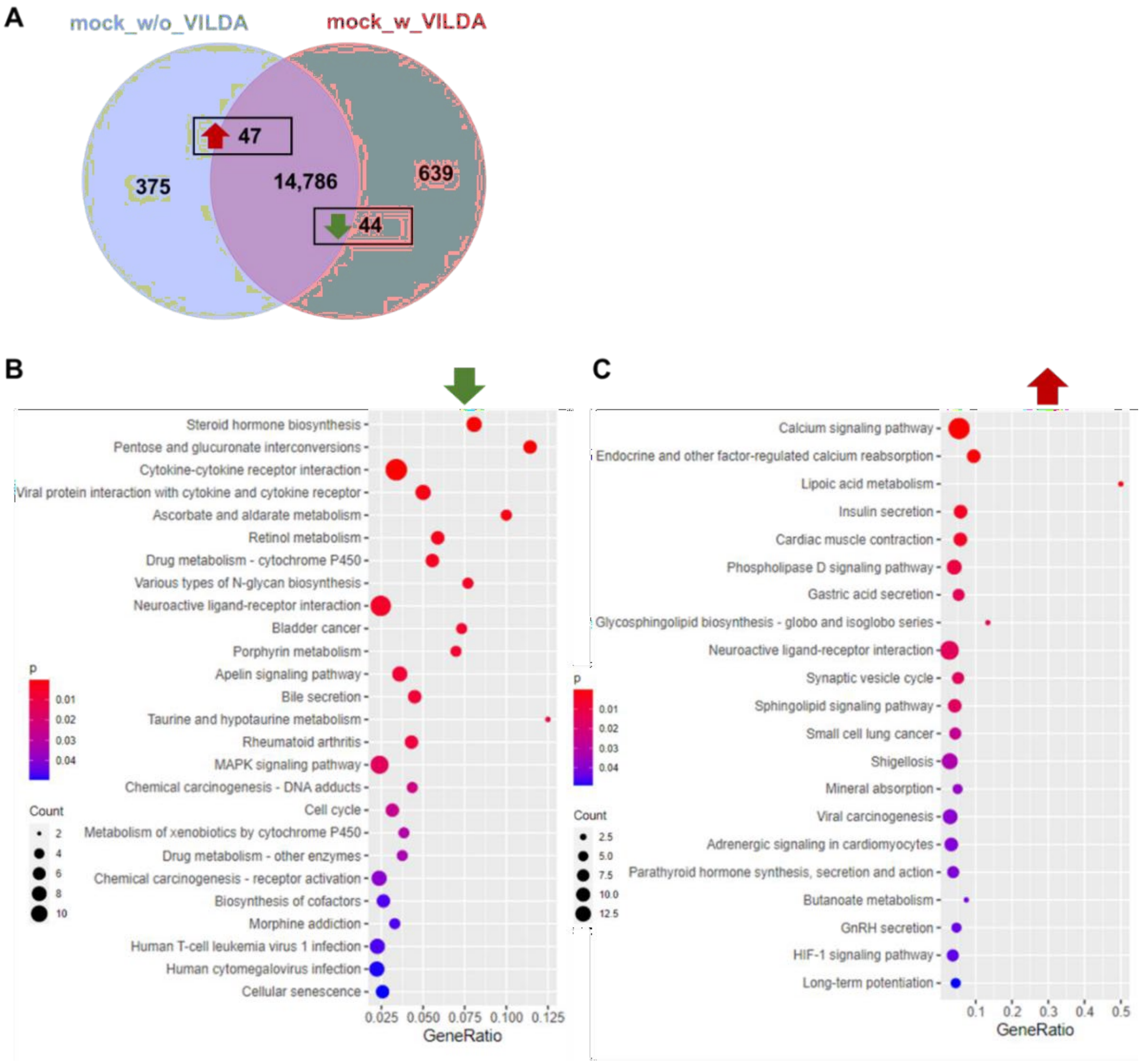

Fig. S11 Global transcriptome analysis of mock-treated HLCs with and without VILDA. (A) Venn diagram of expressed genes upon mock treatment, indicating 14,786 commonly expressed genes. 375 and 639 genes were exclusively expressed w/o or w VILDA, respectively. Among the exclusive and common gene sets, 47 genes were significantly up-, while 44 genes were significantly downregulated upon VILDA. **(B)** KEGG-associated pathway analysis of downregulated gene expression in HLCs treated with mock w and w/o VILDA. **(C)** KEGG-associated pathway analysis of upregulated gene expression in HLCs treated with mock w and w/o VILDA.

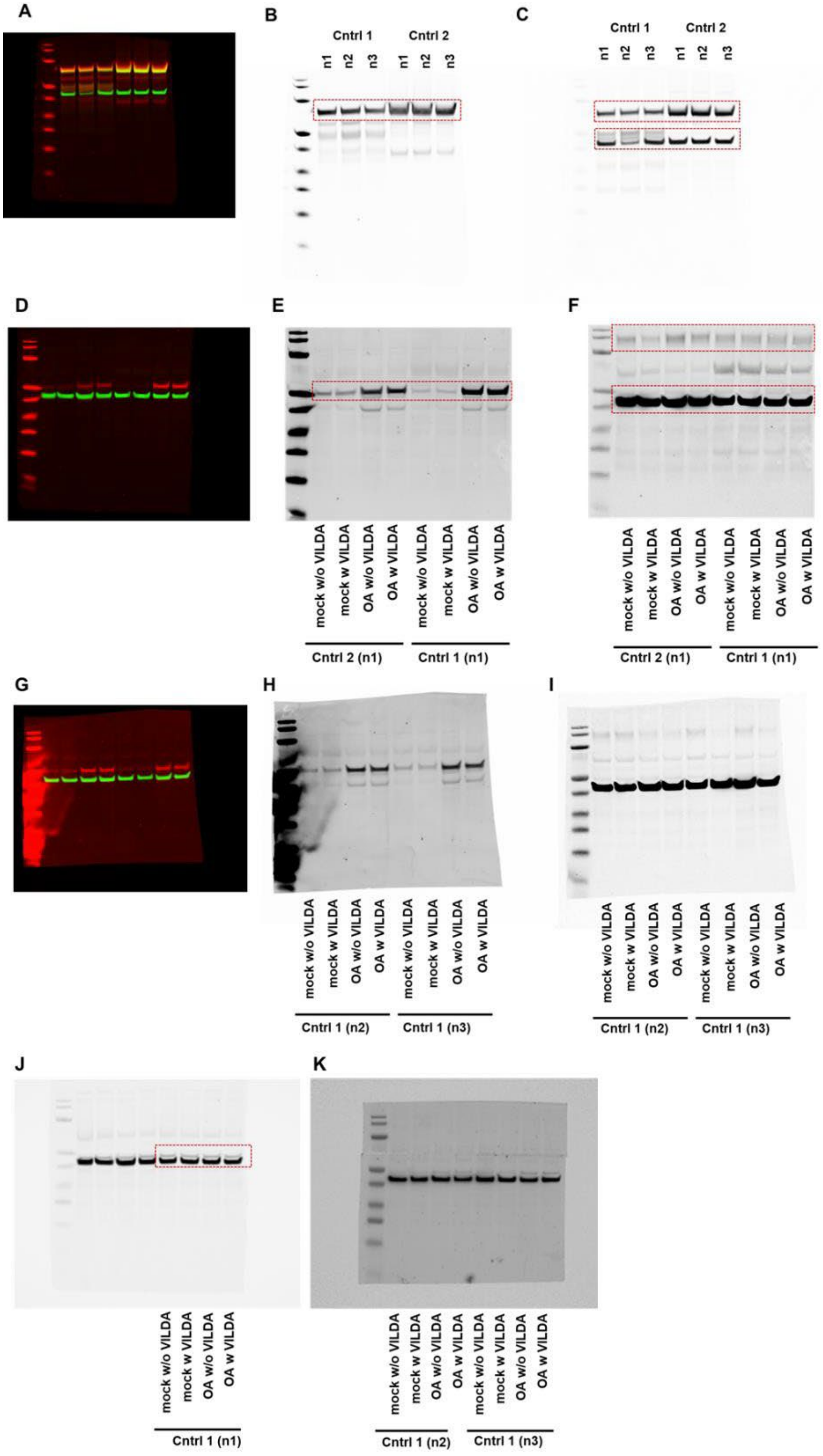

Fig. S12 Uncropped full-length western blot membranes. **(A-C)** WB analysis of HLC differentiation, Fig. 1C. Peqlab peqGOLD Protein marker IV (VWR) was used to estimate the

molecular weight of proteins. Bands (from bottom to top) represent proteins of the following weight in kDa: 10, 15, 25, 35, 40, 55, 100, 130, 170. **(A)** composite membrane detected in 680RD and 800RD channels. **(B)** AFP staining (1:2000 overnight, at 4 °C) and detection with IRDye 680RD goat anti-Rabbit (Licor) 1:10000, 1 h at RT. **(C)** ALB (1:5000 overnight, 4 °C) and bActin (1:5000, 4 °C) staining, detected with IRDye 800RD goat anti-Mouse (Licor) (1:10000, 1 h, RT). Dotted lines indicate the cropping. **(D-F)** Uncropped WB membrane for Fig. 5B: Analysis of HLCs treated with OA w and w/o VILDA n1. **(D)** Composite membrane detected in 680RD and 800RD channels. **(E)** PLIN2 staining (1:2000 overnight, at 4 °C) and detection with IRDye 680RD goat anti-Rabbit (Licor) 1:10000, 1 h at RT. **(F)** DPP4 (1:5000 overnight, 4 °C) and bActin (1:5000, 4 °C) staining, detected with IRDye 800RD goat anti-Mouse (Licor) (1:10000, 1 h, RT). Dotted lines indicate the cropping. **(G-I)** Full-length WB membrane for Fig. 5C: Analysis of Cntrl 1 HLCs treated with OA w and w/o VILDA n2 and n3. **(G)** Composite membrane detected in 680RD and 800RD channels. **(H)** PLIN2 staining (1:2000 overnight, at 4 °C) and detection with IRDye 680RD goat anti-Rabbit (Licor) 1:10000, 1 h at RT. **(I)** DPP4 (1:5000 overnight, 4 °C) and bActin (1:5000, 4 °C) staining, detected with IRDye 800RD goat anti-Mouse (Licor) (1:10000, 1 h, RT). **(J-K):** Uncropped WB membrane for Fig. 6G: Analysis of HLCs treated with OA w and w/o VILDA. PLIN3 Staining (1:5000 overnight, at 4 °C) and detection with IRDye 800RD goat anti-Mouse (Licor) (1:10000, 1 h, RT).

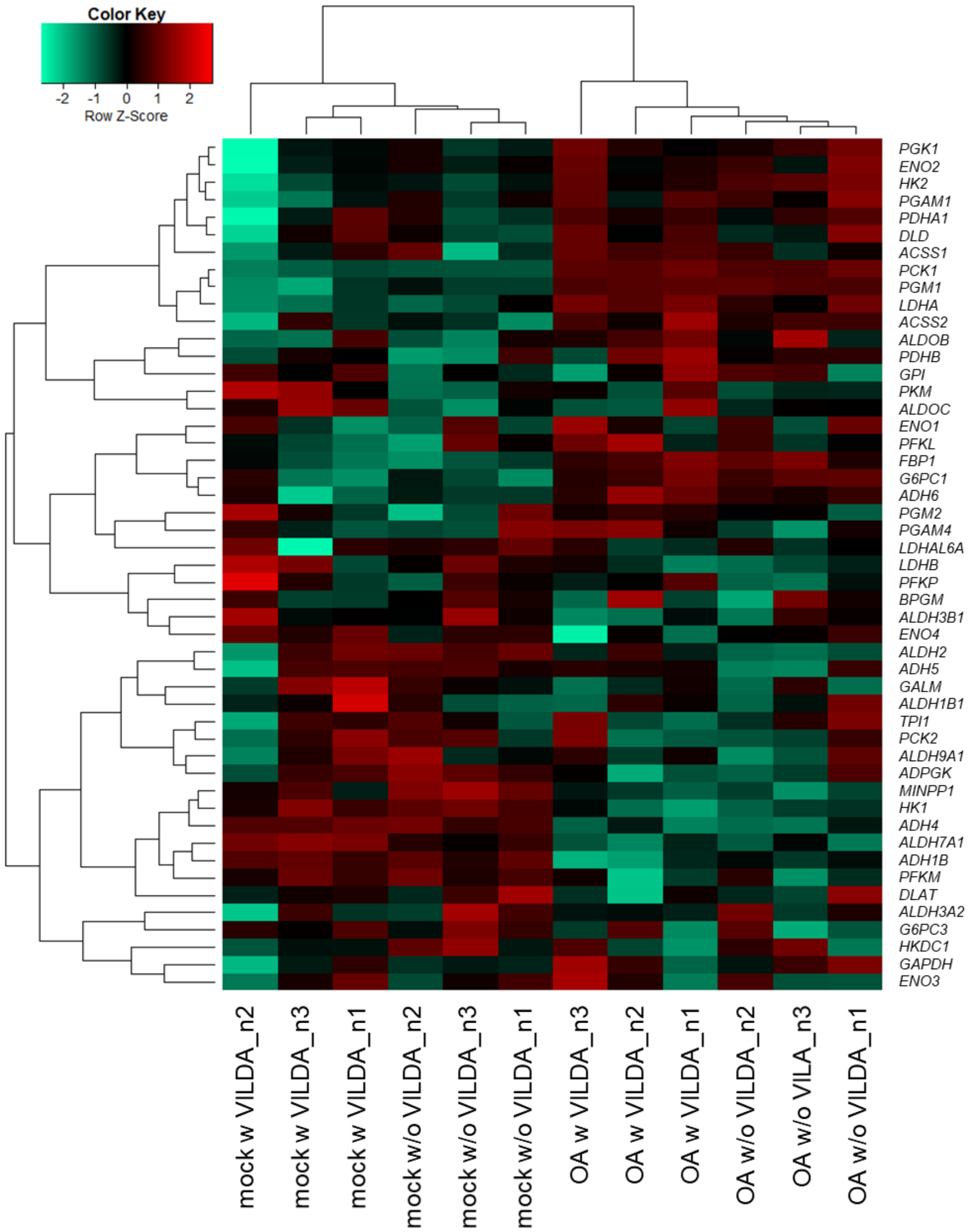

Fig. S13 Person's correlation heatmap analysis of genes of the gluconeogenesis pathway. Pearson's correlation heatmap analysis of genes involved in KEGG-associated pathways of gluconeogenesis.

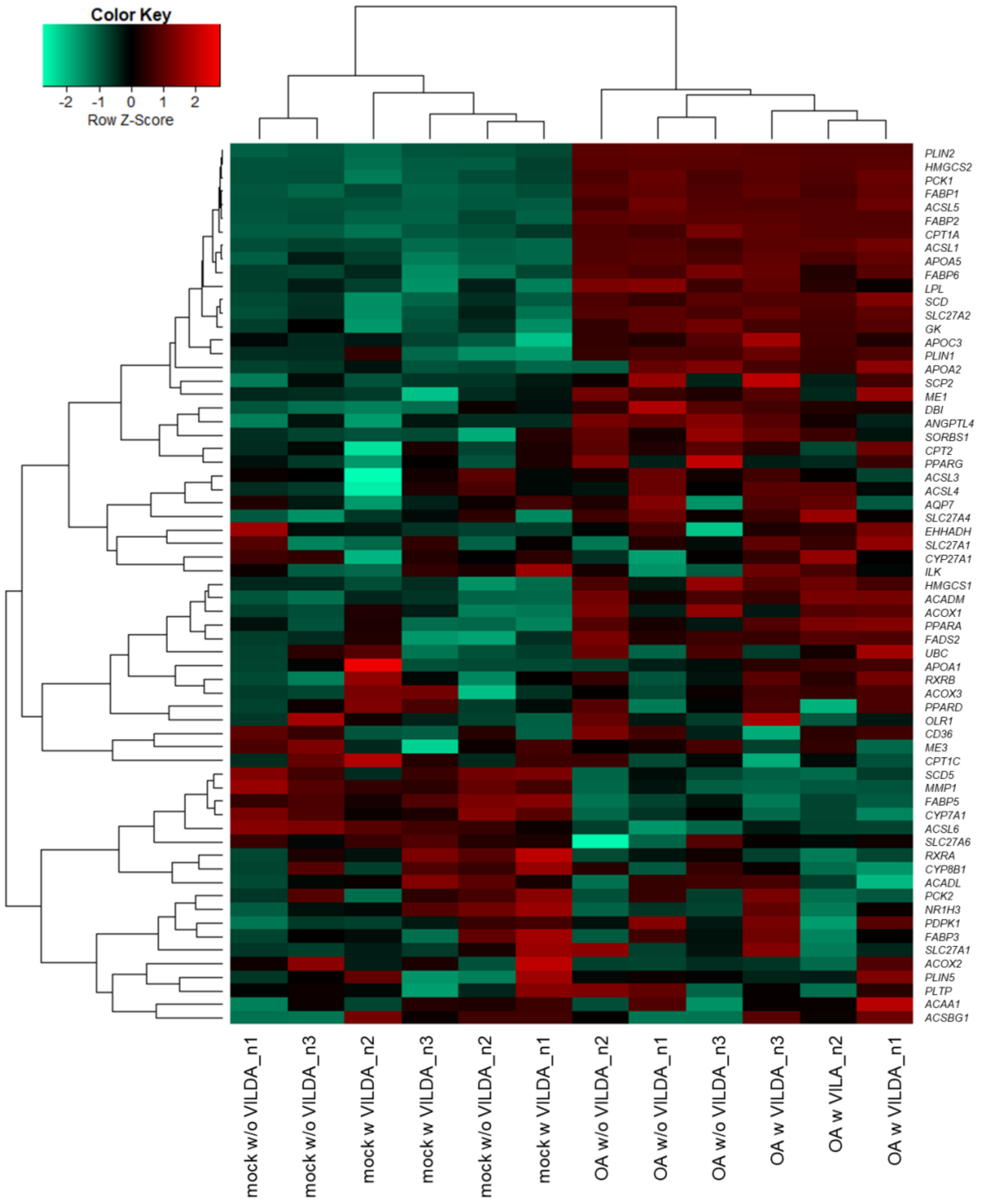

Fig. S14 Person's correlation heatmap analysis of genes of the PPAR signaling pathway. Pearson's correlation heatmap analysis of genes involved in KEGG-associated pathway of PPAR signaling.

4 Supplementary Tables

4.1 Table S1: List of Primers

**Primers**		
**Gene**	**Sequence**	
*	**Forward 5`-3`**	**Reverse 5`-3`**
AFP	AGCAGCTTGGTGGTGGATGA	CCTGAGCTTGGCACAGATCCT
AGPAT2	GGGGCGTCTTCTTCATCA	TTGAGGTTCTCCCTGACCAT
Albumin	AGCTGTTATGGATGATTTCGCAG	CCTCGGCAAAGCAGGTCTC
ANPEP	TGAGCTGTTTGACGCCATCT	GCCCTGCTTGAATACGTCCT
CCL4	GCTAGTAGCTGCCTTCTGCT	CCACAAAGTTGCCAGGAAGC
CPT1A	CCTACCACGGGTGGATGTTC	CAACATGGGTTTTCGGCCTG
CYP2D6	TTCCTGCCTTTCTCAGCAGG	GCACAAAGCTCATAGGGGGA
CYP3A4	GTGACTTTGCCCATTGTTTAGAA AG	CAGGCGTGAGCCACTGTG
DPP4	GTTCTTCTGGGACTGCTGGG	GCTGTAGCATCATCTGTGCCT
FABP1	ATCGTGCAGAATGGGAAGCA	CCCCTGTCATTGTCTCCAGC
FOXA2	TTCAGGCCCGGCTAACTCTG	CCTTGCGTCTCTGCAACACC
HLA-DMA	GGGTTTCCTATCGCTGAAGTG	CCAATAGGCAATTGCTGTGTA
IGFBP1	ACCATCACTTGCCCAGAGTT	AGGAGCAGTACCAGCCAGAC
IL1B	TGTACCTGTCCTGCGTGTTG	ACTGGGCAGACTCAAATTCCA
OCT4	AGTTTGTGCCAGGGTTTTTG	ACTTCACCTTCCCTCCAACC

PCK1	GGGAGTCTCCGGAAGGTGTT	CATGGCAAAGGGGTCATGC
PLIN2	GCTGAGCACATTGAGTCACG	TGGTACACCTTGGATGTTGG
RPLP0	TCGACAATGGCAGCATCTAC	ATCCGTCTCCACAGACAAGG
sHLA-DMB	CGGCCACCATCTGTGCAAGT	CCAGTCCCGAACGATGGGCT
sHLA-DQA1	GAAGGAGACTGCCTGGCG	CATGATGTTCAAGTTGTGTTTTGC
sHLA-DRB1	ACCCAAGCGTGACAAGCCCT	CCCGACTCCACTCAGCATCTTG
SOX17	ACGTGTACTACGGCGCGATG	CTGGTGCTGGTGCTGGTGTT
VCAM1	CGAACCCAAACAAAGGCAGAGTA	GAGGAAGGGCTGACCAAGACG

4.2 Table S2: List of Antibodies

Use:				Western Blot		Immunocytochemistry	
**Antibody**	**Host species**	**Manufacturer**	**Catalog number**	**Dilution**	**Diluent/ Blocking**	**Dilution**	**Diluent/Blocking**
Albumin (ALB)	mouse	Sigma	A6684-.2ml	1:5000	5 % Milk TBS-T	1:100	10 % goat serum
Alpha-fetoprotein (AFP)	rabbit	Sigma	HPA023600	1:2000	5 % Milk TBS-T	1:300	3 % BSA
Angiotensin converting enzyme 2 (ACE2)	goat	R&D Systems	AF933	1:5000	5 % Milk TBS-T	NA	NA
beta-Actin	mouse	Cell Signaling Technologies	3700S	1:5000	5 % Milk TBS-T	NA	NA
Dipeptidyl peptidase 4 (DPP4)	mouse	Proteintech	68383-1-Ig	1:5000	5 % Milk TBS-T	1:100	3 % BSA
E-Cadherin (ECAD)	goat	Cell Signaling Technologies	3195	NA	NA	1:100	3 % BSA
Hepatocyte nuclear factor 4alpha (HNF4a)	rabbit	Abcam	92378	NA	NA	1:250	3 % BSA

Octamer-binding transcription factor 4 (OCT4)	rabbit	Cell Signaling technologies	2840S	NA	NA	1:400	3 % BSA
Perilipin-2 (PLIN2)	rabbit	Proteintech	15294-1-AP	1:2000	5 % Milk TBS-T	1:200	3 % BSA
Perilipin-3/TP47 (PLIN3)	mouse	Proteintech	66523-1-Ig	1:5000	5 % Milk TBS-T	NA	NA
Sry box transcription factor 17 (SOX17)	goat	R&D Systems	AF1924	NA	NA	1:50	3 % BSA
IRDye680RD Goat anti-Rabbit	goat	Licor	925-68070	1:10,000	5 % Milk TBS-T	NA	NA
IRDye800RD<br><br>Goat-anti-Mouse	goat	Licor	925-68071	1:10,000	5 % Milk TBS-T	NA	NA
Alexa 594 gt anti ms IgG (H+L)	goat	Life technologies	A10521	NA	NA	1:500	3 % BSA
Alexa647 gt anti-Rabbit IgG (H+L)	goat	Life technologies	A32733	NA	NA	1:500	3 % BSA
Alexa488 gt anti-Rabbit IgG (H+L	goat	Life technologies	A11008	NA	NA	1:500	3 % BSA
Alexa555 dk anti-Goat IgG	donkey	Life technologies	A32816	NA	NA	1:500	3 % BSA

5

6 References

1. Kim D, Langmead B, Salzberg SL. HISAT: a fast spliced aligner with low memory requirements. Nat Methods 2015;12:357-360.

2. Baruzzo G, Hayer KE, Kim EJ, Di Camillo B, FitzGerald GA, Grant GR. Simulation-based comprehensive benchmarking of RNA-seq aligners. Nat Methods 2017;14:135-139.

3. Li H, Handsaker B, Wysoker A, Fennell T, Ruan J, Homer N, Marth G, et al. The Sequence Alignment/Map format and SAMtools. Bioinformatics 2009;25:2078-2079.

4. Liao Y, Smyth GK, Shi W. featureCounts: an efficient general purpose program for assigning sequence reads to genomic features. Bioinformatics 2014;30:923-930.

5. Law CW, Chen Y, Shi W, Smyth GK. voom: Precision weights unlock linear model analysis tools for RNA-seq read counts. Genome Biol 2014;15:R29.

6. Smyth GK. Linear models and empirical bayes methods for assessing differential expression in microarray experiments. Stat Appl Genet Mol Biol 2004;3:Article3.

7. Chen H, Boutros PC. VennDiagram: a package for the generation of highly-customizable Venn and Euler diagrams in R. BMC Bioinformatics 2011;12:35.

8. Storey JD. A direct approach to false discovery rates. Journal of the Royal Statistical Society: Series B (Statistical Methodology) 2002;64:479-498.
